# Evaluating salivary MMP-8 as a biomarker for periodontal diseases: A systematic review and meta-analysis

**DOI:** 10.1016/j.heliyon.2024.e40402

**Published:** 2024-11-14

**Authors:** Zsuzsanna Domokos, Fanni Simon, Eszter Uhrin, Bence Szabó, Szilárd Váncsa, Gábor Varga, Péter Hegyi, Beáta Kerémi, Orsolya Németh

**Affiliations:** a Department of Community Dentistry, Semmelweis University, Budapest, Hungary; b Centre for Translational Medicine, Semmelweis University, Budapest, Hungary; c Institute for Translational Medicine, Medical School, University of Pécs, Pécs, Hungary; d Institute of Pancreatic Diseases, Semmelweis University, Budapest, Hungary; e Department of Oral Biology, Semmelweis University, Budapest, Hungary; f Department of Restorative Dentistry and Endodontics, Semmelweis University, Budapest, Hungary

**Keywords:** Periodontal disease, MMP-8, Salivary MMP-8, Gingivitis, Prevention, Meta-analysis, Systematic review, Periodontitis

## Abstract

**Objective:**

Periodontitis is the irreversible destructive process of the periodontium and is the major cause of tooth loss in adults worldwide. Diagnosis of early-stage periodontal disease is crucial for improving outcomes, and to this end, the application of indicator biomarkers is gaining interest. One such method involves measuring the level of matrix metalloproteinase-8 (MMP-8). This systematic review and meta-analysis aims to evaluate the salivary MMP-8 level of periodontitis and gingivitis cases compared to healthy controls.

**Data:**

We evaluated all studies that compared different laboratory techniques for measuring salivary MMP-8 levels in periodontitis and gingivitis patients, alongside healthy controls.

**Sources:**

The systematic search was performed on 10 October 2022 in three electronic databases.

**Study selection results:**

Pooled mean differences (MD) were calculated with 95 % confidence intervals (CIs) between the researched groups. In addition, the correlation coefficient results between MMP-8 values and other clinical parameters were narratively summarized.

Based on 20 eligible studies (n = 1725), patients with periodontitis presented significantly higher MMP-8 levels (MD = 273.26 ng/ml, CI: 194.42; 352.10). Similarly, patients with gingivitis presented significantly higher salivary MMP-8 levels than healthy individuals (MD = 122.82 ng/ml, CI: 64.19; 181.45) based on the results of 10 eligible studies (n = 704). Additionally, we found higher MMP-8 levels in periodontitis compared to gingivitis (MD = 112.04 ng/ml, CI: 56.15; 167.92). The correlation results suggest that salivary MMP-8 is associated with different clinical periodontal parameters.

**Conclusion:**

Salivary MMP-8 level measurement may be a reliable method to distinguish between periodontal health and periodontal disease and also to distinguish between gingivitis and periodontitis.

**Clinical significance:**

The measurement of salivary MMP-8 levels may have the potential to differentiate between periodontal health and disease reliably. Accordingly, it can be considered for integration into routine dental examinations as a quick and convenient method for the early detection and prevention of periodontitis, pending further validation.

## Abbreviations:

MMP-8: matrix metalloproteinase-8
CI: confidence interval
MD: mean difference
GCF: gingival crevicular fluid
IFMA: time-resolved immunofluorometric assay
ELISA: enzyme-linked immunosorbent assay
CAL: clinical attachment loss
BOP: bleeding on probing
PPD: probing pocket depth
aMMP-8: active-matrix metalloproteinase-8
tMMP-8: total-matrix metalloproteinase-8
PoC: point of care
IL-1β: interleukin-1β
IL-6: interleukin-6
IL-8: interleukin-8
TNF-α: tumor necrosis factor-α
TIMP: tissue inhibitors of MMP
OPG: osteoprotegerin
CRP: C-reactive protein
ALP: alkaline phosphatase
AST: aspartate aminotransferase

## 1 Introduction

Periodontal disease is the inflammatory destruction of the periodontal tissues [1]. Gingivitis is the inflammatory response of the gingival tissues caused by the accumulation of bacterial biofilm due to inadequate oral hygiene [2]. It is highly prevalent and affects between 50 % and 90 % of adults globally [3]. Though the process is reversible with effective oral hygiene and plaque control [4], if left untreated, gingivitis can prograde into periodontitis in a susceptible host. Periodontitis is an irreversible process characterized by polymicrobial infection-induced inflammation, leading to the loss of supporting connective tissue and alveolar bone [5,6]. Severe periodontitis is the major cause of tooth loss in the adult population, affecting approximately 11 % of adults worldwide, numbering 743 million individuals [7,8]. Early diagnosis is crucial to improving outcomes in this population.

Despite the clinical and radiological diagnostic tools available, diagnosing early-stage periodontal disease and detecting its progression can be challenging [9].

Several saliva biomarkers have been identified as potential indicators of periodontal disease, including MMPs, which play a main role in tissue remodeling, physiological development, malignant tissue destruction, and pathological inflammatory processes [[10], [11], [12]]. Particular MMPs, including MMP-8, MMP-9, and MMP-13, are elevated in the saliva of patients with periodontitis [[13], [14], [15]]. MMP-8, also referred to as collagenase-2, is the primary collagenase in the gingival connective tissue in periodontitis cases and accounts for 90–95 % of the collagenolytic activity in gingival crevicular fluid (GCF), highlighting its critical role as an indicator of periodontitis [11,12,[14], [15], [16], [17], [18], [19], [20], [21]].

Periodontitis begins with the colonization of pathogenic bacteria within the dental biofilm, leading to an inflammatory response in the gingival tissues [22]. This response involves the release of MMP-8 by activated immune cells such as neutrophils [[23], [24], [25]]. As an enzyme that cleaves type I collagen—the main component of the extracellular matrix in the periodontium—MMP-8 is pivotal in the degradation of the collagen matrix, resulting in tissue destruction and attachment loss [24,26]. MMP-8 is released in latent forms from neutrophils and becomes activated during the inflammatory process [27].

The increase of MMP-8 level in inflamed gingival tissue appears in gingival crevicular fluid (GCF) samples, a serum-like fluid that contains a variety of proteins, enzymes, and inflammatory mediators. It can also be detected in mouthrinse and salivary samples, as saliva is secreted from major and minor salivary glands. However, MMP-8 also has non-glandular sources; such as, the aforementioned GCF [13,17,19,20,[28], [29], [30], [31], [32], [33]]. The use of saliva is a non-invasive approach to fluid diagnostics not only in oral diseases but also in systemic diseases [34].

The level of MMP-8 can be measured effectively by both laboratory methods and chair-side tests, which utilize the same monoclonal antibodies [17]. While the criterion standard is the laboratory time-resolved immunofluorometric assay (IFMA) and enzyme-linked immunosorbent assay (ELISA) [11,[35], [36], [37]], there are also chair-side tests available, both qualitative methods providing positive or negative results [38] or quantitative methods [39] providing numeric results [11,40]. Besides mouthrinse-based rapid tests, salivary MMP-8 level measurement presents a promising option in periodontology [41,42].

ELISA and other immunodetection techniques identify both latent and active forms of MMP-8, altogether total-MMP-8 (tMMP-8). In contrast, IFMA can detect active forms of neutrophil and fibroblast MMP-8 isotypes [11,35,37,43].

As the new periodontal classification stands, clinically healthy gingiva shows no color or bleeding on probing changes [44]. However, during this stage, increases in MMP-8 level can be an early biomarker of gingival inflammation.

The present systematic review and meta-analysis aimed to determine if MMP-8 can distinguish between periodontal health and gingivitis, between periodontal health and periodontitis, and between periodontitis and gingivitis. To answer this question, we conducted an analysis to ascertain whether there are significant differences in the salivary MMP-8 levels among periodontitis, gingivitis and healthy cases.

## 2 Materials and methods

### 2.1 Protocol and registration

Our systematic review and meta-analysis was reported following the PRISMA guideline [45] (Supplementary Table 1), following the recommendations of the Cochrane Handbook [46]. The protocol was registered in the International Prospective Register of Systematic Reviews (PROSPERO) in advance (registration number: CRD42022362761).

### 2.2 Information sources and search strategy

The systematic search was performed on 10 October 2022 in three databases: EMBASE, MEDLINE (via PubMed), and the Central Cochrane Register of Controlled Trials (CENTRAL). The search key used in the search process is detailed in Supplementary Table 2. No filters were applied during the search. In addition, the reference lists of selected articles and relevant reviews were also screened to identify additional eligible studies.

### 2.3 Eligibility criteria

In the present meta-analysis, only studies that measured MMP-8 levels from saliva were assessed, while studies measuring the level from the gingival crevicular fluid (GCF) or serum were excluded. The main study question was: How much higher are the salivary MMP-8 levels in periodontitis/gingivitis cases compared to healthy controls? The Population (P) Exposure (E) Comparison (C) Outcome (O) framework was formulated accordingly, to answer the main study question. Eligible studies compared adult (age >18 years) patients with gingivitis or periodontitis to patients with healthy periodontium and assessed the salivary MMP-8 levels.

The second study objective was to assess how MMP-8 level elevation correlates with the conventionally measured clinical periodontal parameters. The Population (P), Prognostic factor (P), and Outcome (O) framework was used for this evaluation. Eligible studies reported the correlation between salivary MMP-8 and Clinical Attachment Loss (CAL), Bleeding of Probing (BOP), Probing Pocket Depth (PPD), or other clinical periodontal outcomes in adult patients.

For the salivary MMP-8 level measurements, any laboratory or chair-side measurement methods were accepted. Only English language cohort and case-control studies were included. Only those studies that contained appropriate data for both case and control groups were included.

### 2.4 Selection process

Endnote X9 was used for the selection process (Clarivate Analytics, Philadelphia, PA, USA). Duplicates were removed both automatically and manually. Then, the articles were selected manually by two independent investigators (ZD and FS) in a stepwise manner based on their title, abstract, and full-text contents. At each selection step, Cohen's kappa coefficient was calculated [47]. In cases of disagreements, a third author was consulted (ON).

In the case of overlapping populations, studies with more participants were included.

If a study included both aggressive and chronic periodontitis populations, the data regarding the chronic periodontitis population was used [48].

### 2.5 Data collection process and data items

Data were manually extracted from each study by two authors independently (ZD and SF). To maintain accuracy, each cross-checked the other's data pool after extraction. Disagreements were solved by consensus. The information was then summarized in a standardized data collection form. The corresponding author was contacted, in the case of missing or incoherent data.

The following data were extracted: first author, the year of publication, country, study design, sample size, mean age of participants, male: female ratio, smoker/non-smoker patients, the definition of periodontitis, gingivitis, and periodontally healthy participants. We extracted the level of salivary MMP-8 in each examined population separately and compared the exposed and control groups. Additionally, a statistical evaluation was carried out to mathematically compare the two exposed groups, based on the results of those studies which provided data for both groups. In addition, the correlation coefficient (r) with the statistical results between MMP-8 and conventionally measured clinical periodontal parameters was extracted.

### 2.6 Study risk of bias assessment

The Newcastle-Ottawa Scale [49] was used to assess the quality of eligible studies. Studies were evaluated based on (1) the selection of study groups, (2) the comparability of groups, (3) and the ascertainment of the exposure. Each study is judged from 0 to 9 stars based on this ranking. Studies with 0–5 stars were considered low-quality, between 6 and 7 to be moderate quality, and between 8 and 9 to be high-quality studies.

The method of recruitment was evaluated for the selection of study groups. It was evaluated whether each group had been chosen only for this study or for another. It was also assessed if groups were matched by, e.g., smoking habits, age, or race.

Two review authors carried out the risk of bias assessment (ZD, FS). If discrepancies occurred, they were solved by a third author (ON).

### 2.7 Synthesis methods and effect measures

Throughout the analyses, the recommendations of Harrer, M., Cuijpers, P., Furukawa, T., & Ebert, D [50]. were followed. All calculations were performed using the open-source R software [51] supplemented with the packages meta [52], dmetar [53] and metafor [54].

Mean differences (MD) were calculated with 95 % confidence intervals (CIs) to compare MMP-8 levels among patient groups using a random effect model with inverse variance weighting. The sample size, mean, and standard deviation (SD) were used for the pooled effect calculation. In those cases where the mean and the SD were not given in the articles, they were estimated using the following measures: standard error (SE), the five main quartiles (minimum, 1st quartile median, 3rd quartile, and maximum), limits of the 95 % confidence intervals (CI). If the studies provided data only separately for subgroups, their results were combined.

For more robust results, a Hartung-Knapp adjustment was applied for the calculations [55]. Between-study heterogeneity was described using Cochrane Q-test and the Higgins & Thompson's I^2^ statistics [56]. Forest plots were used to summarize the results graphically. Studies with conversion are marked on the forest plots with an asterisk. If applicable, that is, if the number of studies in a given analysis was larger than 4, then prediction intervals of results were also included on the forest-plots. For analysis with at least 10 studies, funnel plots and Egger's test with a 10 % significance level were used to check for potential publication bias. Subgroup analyses were performed using mixed-effects models to compare the different salivary MMP-8 measurement methods. The differences between the subgroups were assessed using a Cochrane Q-test, with a 5 % significance level.

Due to the lack of data and heterogeneity, the correlation coefficient results between MMP-8 values and other clinical parameters were narratively summarized.

### 2.8 Additional analysis

The Grading of Recommendations Assessment, Development, and Evaluation (GRADE) framework was used to evaluate the level of certainty of evidence for the outcomes examined in the meta-analysis [57]. The quality of evidence for each outcome was categorized as high, moderate, low, or very low based on the evaluated domains.

## 3 Results

### 3.1 Search and selection

**Fig. 1:**
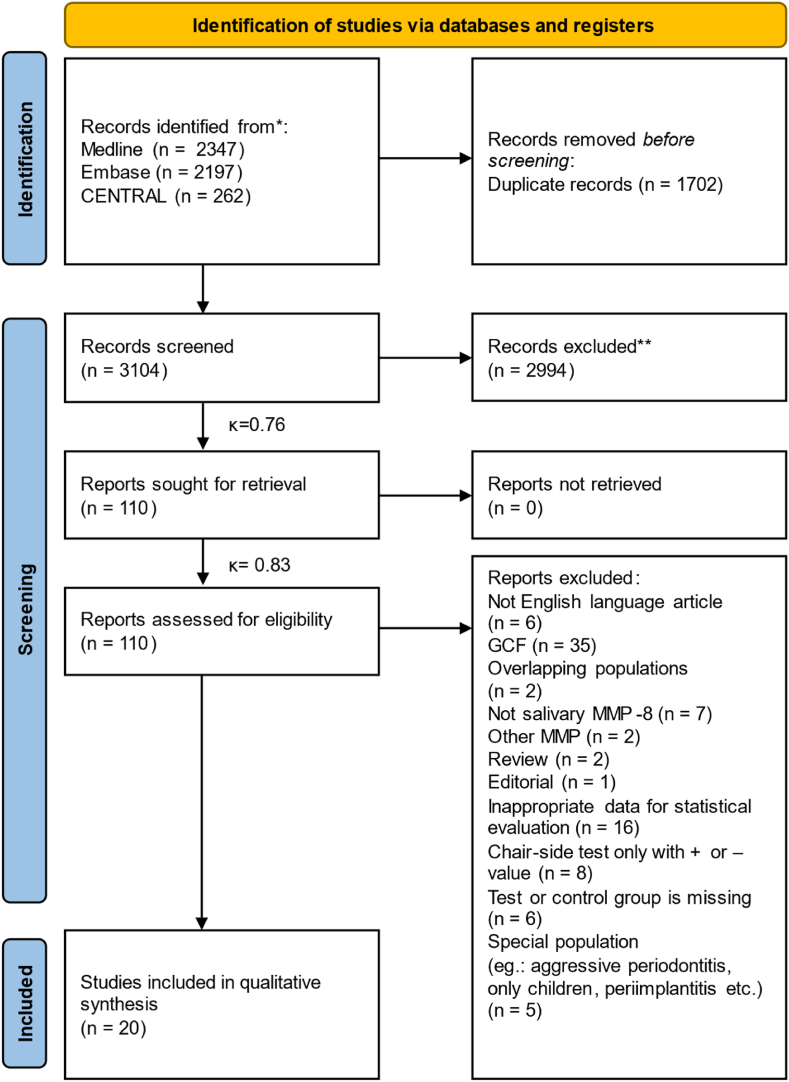
Selection process. *From:* Page MJ, McKenzie JE, Bossuyt PM, Boutron I, Hoffmann TC, Mulrow CD et al. The PRISMA 2020 statement: an updated guideline for reporting systematic reviews. BMJ 2021; 372:n71. https://doi.org/10.1136/bmj.n71.

The search query yielded a total of 4806 records, which was reduced to 3104 after the removal of duplicates. After the title and abstract screening of these records, 110 studies remained for full-text screening. Finally, 20 studies were found eligible for quantitative synthesis (Fig. 1).

### 3.2 Basic characteristics of included studies

Table 1 and Supplementary Table 3 summarize the basic characteristics of the included studies [24,30,36,[58], [59], [60], [61], [62], [63], [64], [65], [66], [67], [68], [69], [70], [71], [72], [73], [74]]. The definition of periodontitis was not homogenous in the included studies. Most studies used the new 2017 classification system; however, some used previous definitions [44]. Gender, age, and smoking habit differences were observed as potential causes of heterogeneity. The method for measuring salivary MMP-8 varied among the studies. Most used the ELISA method or IFMA, while only two used Luminex.

The number of studies using the chair-side method for quantitative measuring was limited; therefore, despite our protocol, a quantitative assessment was not possible.

### 3.3 Risk of bias assessment and publication bias

Supplementary Tables 4 and 5 present the results of the risk of bias assessment. The results suggest that the methodology of the included studies is of high or moderate quality. In most cases, the selection of controls caused bias. According to Egger's test and funnel plot analysis of all included studies, the detection of publication bias is not justified. However, results should be treated with caution (Supplementary Figs. 1–9).

### 3.4 Results of individual studies and synthesis

Table 1 presents the basic characteristics of the studies included in our work, while the discussion section provides a detailed analysis of the findings. The detailed data used for the statistical evaluation are available in Supplementary Tables 6 and 7

**Table 1:** Basic characteristics of included studies.

Study	Country	Sample size (P/G/H)	Mean age	Female:male ratio	Smokers/non-smokers	Method for diagnosing MMP-8
Akbari et al., 2013 [58]	India	100/100/50	30–39 years	ND	mixed cohort	ELISA
Bostanci et al., 2021 [59]	Turkey	60/31/36	P: 39.6 ± 5.7 G:33.1 ± 5.9 H:33.7 ± 6.7	female: male- 71:56	non-smoker	ELISA
Christodoulides et al., 2007 [60]	United States of America	28/-/28	≥18 years of age	ND	not defined	ELISA
Ebersole et al., 2013 [30]	United States of America	50/-/30	P: age: 43.0 ± 10.8 H: age: 31.4 ± 6.8	P: female: 28 % H: female: 46,7 %	mixed cohort	ELISA
Gupta et al., 2015 [62]	India	40/-/20	age range of 35–55 years Group I: 43.30 ± 8.64 Group II: 42.80 ± 8.02 Group III: 44.20 ± 7.40	Group I: 10 male: 10 female Group II: 11 male: 9 female Group III: 14 male: 6 female	mixed cohort	ELISA
Gursoy et al., 2010 [24]	Finland	84/-/81	periodontitis smoker: 48.6 ± 5.3 periodontitis non-smoker: 50.7 ± 4.9 control smoker: 44.4 ± 4.3 Control non-smoker: 48.6 ± 5.7	periodontitis smoker: 52.3 % men periodontitis non-smoker: 67.5 % men control smoker: 42.9 % men Control non-smoker: 33.3 % men	mixed cohort	ELISA, IFMA
Lee et al., 2020 [64]	South Korea	93/-/28	H: 30.04 ± 8.79 P- PS-I: 35.00 ± 15.10 PS-II:49.21 ± 16.92 PS-III: 58.17 ± 14.40 PS-IV: 61.41 ± 11.35	38 male (30.4 %) and 87 female (69.6 %)	mixed cohort	ELISA
Miller et al., 2006 [66]	United States of America	28/-/29	H: age 43.1 ± 7.2 P: age 45.4 ± 8.5	H: 41.4 % male P: 42.9 % male	mixed cohort	ELISA
Rai et al., 2008 [70]	India	20/18/15	P: 35.3 ± 9.6 G: 36.1 ± 9.3 H: 35.1 ± 8.7	ND	–	ELISA
Ramseier et al., 2009 [71]	United States of America	49/32/18	Group A (healthy): 45 years, Group B (gingivitis): 42 years, Group C (mild chronic periodontitis): 53 years, Group D (moderate to severe chronic periodontitis): 50 years	Group A (healthy): 56 % (males %) Group B (gingivitis): 41 % Group C (mild chronic periodontitis): 39 % Group D (moderate to severe chronic periodontitis): 38 %	mixed cohort	ELISA
Rangbulla et al., 2017 [72]	India	30/-/20	aged 18–45 years	ND	non-smokers	ELISA
Umeizudike et al., 2022 [73]	United Kingdom	67/63/59	40.4 ± 11.7 years (range 18–62 years)	Females (n = 118) represented 62.4 % of the total study population	non-smokers	ELISA, IFMA
Zhang et al., 2021 [74]	China	31/24/25	P: 42.58 ± 3.39 G: 26.32 ± 4.02 H: 24.68 ± 3.52	P: 17/14 (M/F) G: 11/13 H: 12/13	non-smokers	ELISA
Keles et al., 2020 [63]	Turkey	40/20/23	aged 25–50 years mean age: 37.16 ± 5.96 years	41 females and 42 males	non-smokers	IFMA
Mauramo et al., 2021 [65]	Switzerland	116/-/86	Mean age: P: 48.2 (29–56) H:42.9 (25–57)	Male/Female (%/%) P:55/61 (47.4/52.6) H:27/59 (31.4/68.6)	mixed cohort	IFMA
Nizam et al., 2014 [67]	Turkey	18 (chronic periodontitis) /-/18	P: GCP age 50.0∗ (45.0–54.0) H: age 44.50∗ (39.50–52.50)	P: GCP: 10 males and 8 females; H: 11 males and 7 females	mixed cohort	IFMA
Noack et al., 2017 [68]	Germany	20/20/19	H: 24.3 (23.6–26.4) G: 24.6 (23.2–25.3) P: 50.3 (39.1–57.6)	H: 3 (15.8) Male (%) G: 11 (55.0) P: 12 (60.0)	mixed cohort	IFMA
Ozturk et al., 2021 [69]	Turkey	37/21/22	PSIV: 41 ± 7.8 PSIII:44.7 ± 9.6 G: 30 ± 9.0 H: 31 ± 6.4	PSIII: 11/8 (Females/Males) PSIV: 8/10 G: 11/10 H: 10/12	non-smokers	IFMA
Ebersole et al., 2015 [61]	United States of America	101/43/65	Age (years; mean ± SD) H: 28.2 ± 5.9 G:27.8 ± 4.5 P:42.0 ± 10.4	H: 60.0 (Female %) G: 48.8 P: 32.7	mixed cohort	LUMINEX
Johnson et al., 2016 [36]	United States of America	31/-/10	P: 44.9 ± 14.3 H: 31.5 ± 5.2	ND	mixed cohort	LUMINEX

Abbreviations: P: periodontitis group, G: gingivitis group, M:male, F:female, H: healthy group, BOP: Bleeding On Probing, PPD: Probing Pocket Depth, CAL: Clinical Attachment Loss, ND: not defined.

#### 3.4.1 MMP-8 level elevation in periodontitis

**Fig. 2:**
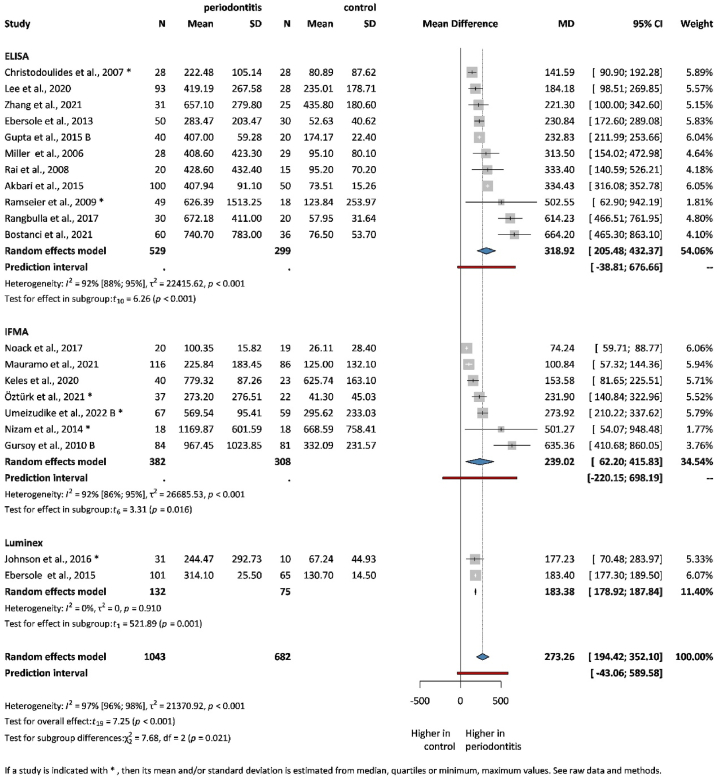
Mean difference of MMP-8 level results in periodontitis patients compared to healthy population.

Fig. 2 summarizes the overall and subgroup differences between patients with a healthy periodontium and those with periodontitis. Two studies measured the MMP-8 level with both ELISA and IFMA [24,73]. Therefore, the overall effect was calculated using both methods. In the evaluation, the IFMA method was used. However, Supplementary Fig. 10 contains the evaluation that used the ELISA methods.

A total of 20 studies were selected for analyses covering 1725 patients. The level of MMP-8 was specified in ng/ml. Patients with periodontitis presented significantly higher salivary MMP-8 levels (MD = 273.26, CI: 194.42; 352.10). The heterogeneity was high, possibly due to the different population characteristics used in the studies and the different laboratory measurement techniques, and is further detailed in the limitations part. When assessing the difference based on the measurement method, MMP-8 levels measured with ELISA, which measures tMMP-8 levels (MD = 318.92, CI: 205.48; 432.37) showed the highest difference, followed by IFMA, which measures aMMP-8 levels (MD = 239.02, CI: 62.20; 415.83) and LUMINEX (MD = 183.38, CI: 178.92; 187.84).

#### 3.4.2 MMP-8 level elevation in gingivitis

**Fig. 3:**
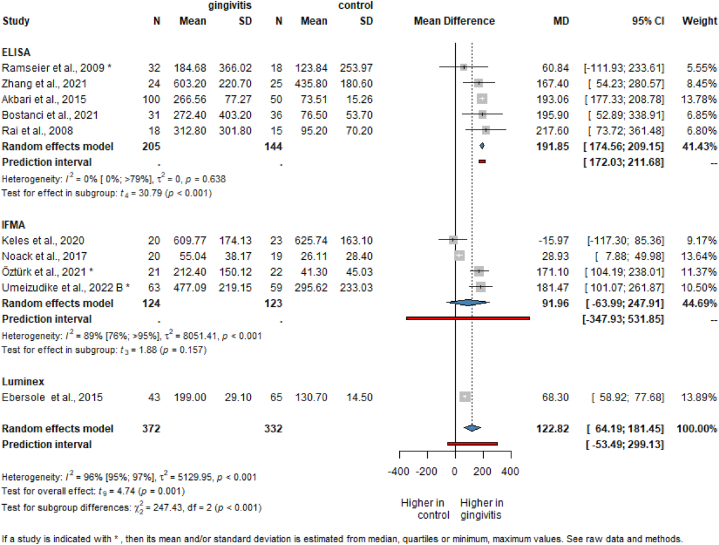
Mean difference of MMP-8 level results in gingivitis patients compared to healthy population.

Our result regarding the gingivitis vs. healthy population comparison is based on the data from 10 studies covering the results of 704 patients (Fig. 3) [58,59,61,63,[68], [69], [70], [71],^73^,^74^].

Patients with gingivitis presented higher salivary MMP-8 levels than healthy individuals (MD = 122.82, CI: 64.19; 181.45). The mean difference in the evaluation measured with ELISA (tMMP-8) was the highest (MD = 191.85, CI: 174.56; 209.15), followed by IFMA (aMMP-8) (MD = 91.96, CI: −63.99; 247.91) and Luminex (MD = 68.30, CI: 58.92; 77.68). The IFMA results do not represent statistically significant results. One study measured the MMP-8 level both by ELISA and IFMA [73]. Supplementary Fig. 11 presents the pooled results using the ELISA method.

#### 3.4.3 MMP-8 level elevation in gingivitis compared to periodontitis

**Fig. 4:**
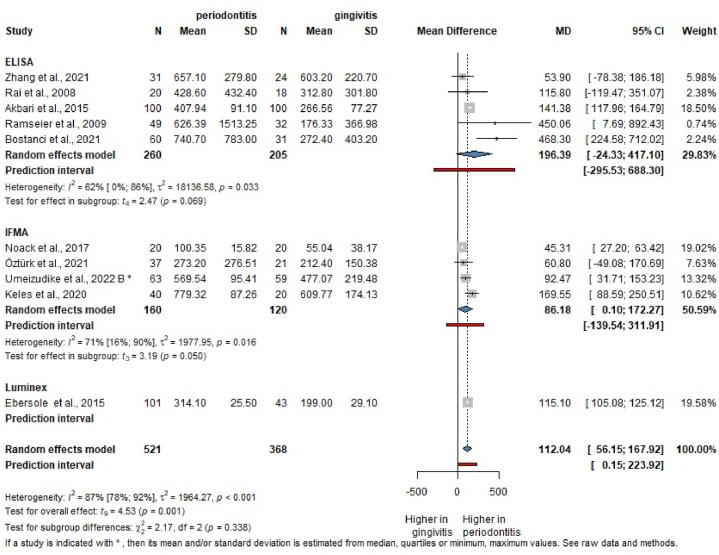
Mean difference in MMP-8 level results in periodontitis population compared to gingivitis patients.

Additionally, the salivary MMP-8 levels were compared between patients with gingivitis and periodontitis. In the group of patients with periodontitis, salivary MMP-8 levels were significantly higher (MD = 112.04, CI: 56.15; 167.92). (Fig. 4). The mean difference in the evaluation measured with ELISA (tMMP-8) was the highest (MD = 196.39, CI: −24.33; 417.10), by IFMA (aMMP-8) it was MD = 86.18, (CI: 0.10; 172.27) and by Luminex one study found MD = 115.10 (CI:105.08; 125.12) Supplementary Fig. 12 contains results using results based on the ELISA measurement [73].

#### 3.4.4 Correlation

The different correlation results retrieved from the studies are summarized in Table 2 [26,62,63,[66], [67], [68],^74^,^75^].

**Table 2:** Correlation coefficients reported in included studies.

Study, year	Salivary MMP-8 level correlated with x	Correlation type	Corr.	p value	Method for diagnosing MMP-8
Noack et al., 2017	PI	Spearmann	0.651	p ≤ 0.01	IFMA
	GI	Spearmann	0.688	p ≤ 0.01	
	mean PPD	Spearmann	0.677	p ≤ 0.01	
	number of sites with PPD≥ 4 mm	Spearmann	0.669	p ≤ 0.01	
	number of sites with PPD≥6 mm	Spearmann	0.682	p ≤ 0.01	
	smoking	Spearmann	0.39	p ≤ 0.01	
Gupta et al., 2015	GI	Pearson	0.65	p < 0.001	ELISA
	PI	Pearson	0.93	p < 0.001	
	PPD	Pearson	0.95	p < 0.001	
	CAL	Pearson	0.94	p < 0.001	
Miller et al., 2006	BOP	Pearson	0.58	p = 0.001	ELISA
	CAL>2 mm	Pearson	0.39	p = 0.003	
	Sites with PPD>4 mm	Pearson	0.62	p < 0.001	
	Sites with PPD>5 mm	Pearson	0.62	p < 0.001	
Nizam et al., 2014	Chronic periodontitis group: PPD	Spearmann	0.61	p = 0.007	IFMA
	Chronic periodontitis group: PI	Spearmann	0.634	p = 0.005	
	Chronic periodontitis group: BOP	Spearmann	0.47	p = 0.049	
	Healthy group: PI	Spearmann	−0.289	p = 0.274	
	Healthy group: age	Spearmann	0.439	p = 0.09	
Zhang et al., 2021	PPD	Spearmann	0.319	p = 0.008	ELISA
	BOP	Spearmann	0.377	p = 0.002	
	Age	Spearmann	0.161	p = 0.187	
Gursoy et al., 2013 (same population as Gursoy 2010) [26]	MMP-9	Spearmann	0.344	p < 0.001	IFMA
	number of teeth with PPD ≥4 mm	Spearmann	0.359	p < 0.001	
	total bone loss	Spearmann	0.192	p = 0.004	
Keles et al., 2020	PPD	Spearmann	0.469	p ≤ 0.1	IFMA
	CAL	Spearmann	0.507	p ≤ 0.1	
	PI	Spearmann	0.382	p ≤ 0.1	
	BOP	Spearmann	0.241	p ≤ 0.5	
Ebersole et al., 2015	Total population: BOP%>0	Pearson	0.484	p ≤ 0.5	Luminex
	Total population: PPD>4 mm	Pearson	0.450	p ≤ 0.5	
	Total population: PPD>5 mm	Pearson	0.459	p ≤ 0.5	
	Total population: Mean PPD	Pearson	0.463	p ≤ 0.5	
	Periodontitis group: BOP%>0	Pearson	0.383	p ≤ 0.5	
	Periodontitis group: PPD>4 mm	Pearson	0.361	p ≤ 0.5	
	Periodontitis group: PPD>5 mm	Pearson	0.375	p ≤ 0.5	
	Periodontitis group: Mean PPD	Pearson	0.386	p ≤ 0.5	
	Gingivitis group: BOP%>0	Pearson	0.258		
	Gingivitis group: PPD>4 mm	Pearson	−0.008		
	Gingivitis group: PPD>5 mm	Pearson	−0.140		
	Gingivitis group: Mean PPD	Pearson	−0.060		
	Healthy group: BOP%>0	Pearson	0.166		
	Healthy group: PPD>4 mm	Pearson	−0.125		
	Healthy group: PPD>5 mm	Pearson	−0.118		
	Healthy group: Mean PPD	Pearson	0.007		

Abbreviations: BOP: Bleeding on Probing, CAL: Clinical Attachment Loss, PPD: Probing Pocket Depth, PI: Plaque Index, GI: Gingival Index.

In most cases, researchers investigated the correlation between PPD and MMP-8 levels. All studies determined positive association; however, the range varied (from r = 0.319, to r = 0.95). The association between salivary MMP-8 level and BOP or CAL was also highly investigated, and a positive association was found, with wide ranges. (mean CAL: from r = 0.507 to r = 0.94; BOP from r = 0.241 to r = 0.58). Besides the previous parameters, the association between salivary MMP-8 and PI, GI, or age was also investigated in the studies. There were studies that examined the correlation between MMP-8 and the aforementioned clinical periodontal parameters separately in the periodontitis, gingivitis, and healthy groups. These studies could only prove a positive association in the periodontitis group [61,67]. In the gingivitis and healthy groups, the periodontal parameters investigated were not associated with salivary MMP-8 level, or only a very weak association was found.

### 3.5 Certainty of evidence

The assessment of the quality of evidence across all three outcomes revealed a moderate level (Supplementary Table 8). The main reason for not classifying them as high certainty was the study design; however randomized control studies were not available and were furthermore not meaningful due to the study question.

## 4 Discussion

### 4.1 Summary of main findings

This study is the first to systematically investigate if salivary MMP-8 can distinguish periodontitis and gingivitis from periodontal health, respectively, and also, distinguish between the two diseases, gingivitis and periodontitis. Furthermore, the number of available publications investigating the topic, mainly in the last few years, reflects the novelty, relevance, and high importance of the topic. Based on our results, salivary MMP-8 level is significantly different in patients with periodontitis, gingivitis and healthy periodontium, with periodontitis cases having the highest and healthy controls having the lowest values. Therefore, salivary MMP-8 measurement may be reliable method for distinguishing gingivitis and periodontitis from healthy periodontium, and for distinguishing between the two different diseases. Nevertheless, exact cut-off values should be investigated to differentiate between the study groups, necessitating a future diagnostic meta-analysis. Regarding the diagnostic value of MMP-8 in periodontitis, Zhang et al. carried out a similar systematic review and meta-analysis but with a slightly different statistical evaluation [76]. However, they did not investigate gingivitis patients, only periodontitis. Also, our review included a higher number of eligible studies, as the applicability of MMP-8 in periodontal practice is gaining increasing interest, thus reinforcing the statistical power and the reliability of the results. Our results regarding periodontitis patients are concordant with the results of Zhang et al. in that the salivary MMP-8 level is significantly higher in periodontitis patients than in healthy populations. Moreover, our analysis confirmed that the MMP-8 level is significantly different in gingivitis compared to periodontal health or periodontitis.

### 4.2 Laboratory-based immunological assays

In this respect, the present study resolves one of the most significant limitations in the previous meta-analysis [76], in which the assessment included all laboratory techniques, resulting in a high heterogeneity.

Currently, laboratory-based immunological assays are acknowledged as the criterion for quantifying salivary biomarkers associated with periodontal disease [26,[77], [78], [79]]. ELISA and similar immunodetection methods measure latent and active variants of MMP-8 (tMMP-8). On the other hand, IFMA specifically detects active forms (aMMP-8) of MMP-8 isotypes [11,35,37,43,80]; therefore, separate handling of two measurement techniques had high priority, making subgroup analyses crucial. Studies suggest that aMMP-8 levels are more precise than tMMP-8 measurements in screening for periodontal disease and are more correlated with disease progression [17,27,35,81,82]. Only one study included in our meta-analysis applied both ELISA (tMMP-8) and IFMA (aMMP-8) methods and reaffirmed that both methods can distinguish periodontal health, gingivitis and periodontitis based on saliva MMP-8 results; however, IFMA can detect changes in periodontal therapy as well [73].

### 4.3 Chair-side method

While laboratory-based techniques are sensitive and specific, these methods are highly expensive and require specially trained staff. As a result, there is an unmet need for a simple but reliable method that can be introduced into routine clinical practice.

Recent investigations have identified a correlation between aMMP-8 levels and the stage of periodontitis, while tMMP-8 has not shown such an association [[83], [84], [85], [86], [87]]. Point of care (PoC)/chair-side tests are also based on aMMP-8 immunoassay and perform successfully in periodontology [36,38,[87], [88], [89], [90], [91], [92], [93], [94]].

Sorsa et al. examined the potential feasibility of integrating aMMP-8 as a diagnostic tool in the latest periodontitis classification of staging and grading system to improve periodontal disease assessment and treatment outcomes. They used aMMP-8 PoC mouthrinse testing and found healthy participants had significantly lower MMP-8 levels than patients with severe periodontitis; accordingly they recommended integrating aMMP-8 PoC/chair-side mouthrinse testing into the new classification system in periodontology [87,95]. It can improve diagnostic accuracy, and it can be used to assess disease progression [44,87]. PoC chair-side tests can detect, predict and monitor the stage and treatment of periodontitis [96].

In our systematic review and meta-analysis, the evaluation of the results of studies using a quantitative chair-side test was also planned. However, only a very few data were available, prohibiting statistical analysis. Nevertheless, there are promising results that necessitate the undertaking of future studies to expand upon the findings.

Currently, the available data on quantitative chair-side tests are insufficient to perform a comprehensive meta-analysis. However, the number of existing studies has allowed for the execution of a diagnostic meta-analysis using PoC chair-side tests, which yield either positive or negative results. Wei et al. [97] concluded that aMMP-8 PoC tests exhibit moderate accuracy to detect periodontitis. The pooled sensitivity and specificity for these tests were reported as follows: using saliva samples, sensitivity was 0.60 (95 % CI: 0.13–0.95) and specificity was 0.72 (95 % CI: 0.12–0.99); for the first oral rinse, sensitivity was 0.75 (95 % CI: 0.26–0.98) and specificity was 0.62 (95 % CI: 0.07–0.98); for the second oral rinse, sensitivity was 0.63 (95 % CI: 0.39–0.83) and specificity was 0.84 (95 % CI: 0.61–0.96). Despite these findings, the analysis was limited to only six eligible studies. Therefore, they emphasized what we also concluded, that there is an urgent need for additional research to enhance the robustness of future meta-analyses.

### 4.4 Samples

As mentioned above, MMP-8 level is elevated in GCF, and thus, in saliva and mouthrinse in cases of periodontitis. For GCF sampling, a paper is inserted into the gingival sulcus [98]. For saliva sampling, the Navazesh protocol is usually followed [99]. Akbari et al. found that the MMP-8 level was higher in GCF samples, compared to saliva samples, since the MMP-s are primarily isolated in GCF and may be expressed in saliva [58,100]. However, saliva and oral rinse sampling is quick and easy and does not require specially-trained staff. Furthermore, it is reliable in terms of specificity and sensitivity. Mouthrinse was found to measure aMMP-8 precisely [101,102], while saliva sampling was also found to be reliable [94]. Deng et al. found that periodontitis patients had significantly elevated aMMP-8 levels and increased test positivity rates in both saliva and oral rinse samples; however, first oral rinse samples were superior to saliva [103]. Nevertheless, introducing the application of oral fluid measurement into clinical practice should be considered [28,58,92,104,105].

It is also known that periodontitis can contribute to systemic inflammation and affects systemic health [106]. Thus, it was demonstrated that the level of MMP-8 rises in serum as well. However, this method is more invasive and not easily accessible. Therefore, it is not recommended for use in everyday dental practice [63,68,107].

More studies investigated the role of MMP-8 level elevation in gingivitis in different samples. Keles et al. found that saliva MMP-8 measurement could differentiate healthy, gingivitis, and periodontitis populations. Moreover, serum and GCF MMP-8 levels were higher in gingivitis and periodontitis than in healthy populations. However, unlike saliva measurement, these methods failed to distinguish between periodontitis and gingivitis [63]. Additionally, other studies investigated different methods with promising results [108]. Mantyla et al. [39] found that both quantitative immunofluorometric assay and MMP-8 dip-stick test could differentiate healthy and gingivitis sites from periodontitis. The aMMP-8 is not synonymous to tMMP-8 in periodontitis diagnostics. Nascimento et al. investigated patients with experimental gingivitis, and a relationship was discovered between MMP-8 and the development of gingivitis as the response to the accumulation of plaque [109].

The association between aMMP-8 and BOP – as the primary indicator for gingivitis – was investigated in the study of Raisananen et al. They found a low but significant correlation between BOP and MMP-8 [110]. Sorsa et al. concluded a strong positive association between BOP and MMP-8 r = 0.586, p < 0.001 in their study using a quantitative aMMP-8 PoC (PoC)/chair-side mouthrinse test [87].

Both qualitative and quantitative PoC or chair-side assay technologies have been developed for the early detection of elevated levels of MMP-8 in oral fluids and serum [17,111].

### 4.5 Perspectives for clinical application in the therapy follow-up

During non-surgical periodontal therapy (including oral hygiene instruction, scaling, and root planing), the reduction of inflammation leads to pocket depth and bleeding decrease. Studies demonstrated that, with the reduction of periodontal pocket depth, full mouth bleeding score, and clinical attachment loss, the level of MMP-8 in GCF decreases [16,112]. More studies demonstrated that aMMP-8 is further associated with and expresses the severity and progression of periodontal disease [33,81,82,113]. Mauramo et al. found that higher levels of MMP-8, both in GCF and saliva, were associated with greater PPD and BOP [114]. After the treatment, MMP-8 levels can be similar to those of healthy individuals [2,13,39,58,112,115]. This decrease in enzyme levels after treatment suggests that non-surgical periodontal therapy can effectively reduce the periodontopathogenic bacteria and can be used to measure treatment efficacy [17].

### 4.6 Biomarkers associated with periodontitis

Other biomarkers are associated with periodontitis. The levels of some inflammatory cytokines such as interleukin-1β (IL-1β), interleukin-6 (IL-6), IL-8, and tumor necrosis factor-α (TNF-α) were found to be elevated. However, a decreased level of the tissue inhibitor of TIMP-1 was observed [[116], [117], [118], [119], [120]]. The salivary levels of certain bacteria, such as *Porphyromonas gingivalis* and *Aggregatibacter actinomycetemcomitans*, have also been found to be elevated and might be used as microbial markers of periodontitis [[121], [122], [123]]. Some studies found elevated levels of Osteoprotegerin (OPG) and C-reactive protein (CRP) [[124], [125], [126]]. Enzymes such as alkaline phosphatase (ALP) and aspartate aminotransferase (AST) have also been shown to be elevated in association with periodontitis [116,[127], [128], [129], [130]].

In their study, Bostanci et al. validated ten biomarkers: MMP-8, -9, -2, -3, TIMP-1, OPG, interleukin-1-beta and −8 (IL-1-β, IL-8), hepatocyte growth factor (HGF) and lipopolysaccharide-binding protein (LBP) [59]. MMP-8 showed the highest significant difference from the investigated biomarkers between periodontitis and healthy controls (ratio: 9.69, p < 0.001) and showed the second highest difference between gingivitis and healthy controls (ratio: 3.56, p = 0.001).

Overall, the use of saliva biomarkers in the diagnosis of periodontitis is an area of active research. Saliva biomarkers can be used to diagnose periodontitis, assess disease severity, and monitor the response to treatment, and the most promising biomarker appears to be MMP-8.

### 4.7 Strengths and limitations

Regarding the strengths of our study, we followed a rigorous methodology throughout the whole process. This was the first study to systematically investigate periodontitis and gingivitis patients' MMP-8 results, with regard to predicting and assessing future periodontal disease severity and providing a basis for measuring oral health. We evaluated the different laboratory methods separately: ELISA, IFMA, and Luminex, to reduce bias caused by this factor. Regarding the risk of bias assessment, we used only high-quality studies for statistical evaluation. The high number of publications investigating the topic, mainly in the last few years, reflects both the novelty and the importance of the topic.

The most significant limitation of our evaluation was the high heterogeneity of studies, which could not be reduced significantly with subgroup analysis. The high heterogeneity might be due to the different study populations. Smoking is the most known risk factor for developing periodontitis [131]. Smoking can worsen the defense against oral microbes and disturb the MMP-8/TIMP-1 ratio, thus masking differences and affecting results [17,24,132]. Definitions of periodontitis and gingivitis varied, so the selection of study participants was heterogeneous. We had also planned to evaluate the results of quantitative chair-side tests; however, only few publications were available and were not suitable for statistical analysis. This highlights the unmet need for more studies assessing the reliability of these novel chair-side devices, which could provide an efficient and convenient yet reliable option in clinical practice.

### 4.8 Implication for practice and research

Assessing the accuracy of MMP-8 level measurement is a highly investigated topic. A large number of publications were available, enabling a reliable statistical evaluation of laboratory methods. However, there are not yet enough publications to assess the chair-side methods.

Saliva MMP-8 level measurement is a rapid, easy, non-invasive method for diagnosing periodontitis. Moreover, the elevation of MMP-8 level appears before clinical signs of irreversible destruction. Therefore, patients can receive the necessary intervention earlier and thus benefit from an accordingly higher chance of positive outcomes.

Though the laboratory methods to evaluate salivary MMP-8 level are accurate, they are expensive, time-consuming, and require specially trained staff. For these reasons, such laboratory methods cannot be implemented into everyday dental practice. In contrast, chair-side methods are cost-effective, quick, and easy to perform. Therefore, they have the potential to be introduced in every dental practice. Besides the saliva-based chair-side test, there are mouthrinse-based rapid tests, which are performing well in clinical practice and generating increasing interest. Accordingly, more studies are needed to assess the accuracy of different chair-side methods. Overall, MMP-8 level measurement has a high potential to set a new foundation and standard for periodontitis prevention.

### 4.9 Conclusions

The salivary MMP-8 level exhibits a significant elevation in individuals diagnosed with periodontitis in contrast to both healthy controls and those affected with gingivitis. Furthermore, individuals with gingivitis demonstrate a higher salivary MMP-8 level compared to healthy controls. Therefore, salivary MMP-8 level measurement may be reliable for differentiating healthy periodontal cases from gingivitis or periodontitis. MMP measurement has a high potential to bring about a breakthrough in the field of periodontology.

## CRediT authorship contribution statement

**Zsuzsanna Domokos:** Writing – review & editing, Writing – original draft, Project administration, Methodology, Formal analysis, Conceptualization. **Fanni Simon:** Writing – original draft, Project administration, Conceptualization. **Eszter Uhrin:** Writing – review & editing, Visualization, Formal analysis, Conceptualization. **Bence Szabó:** Writing – review & editing, Visualization, Validation, Investigation. **Szilárd Váncsa:** Writing – review & editing, Supervision, Methodology, Conceptualization. **Gábor Varga:** Writing – review & editing, Supervision, Methodology, Conceptualization. **Péter Hegyi:** Writing – review & editing, Supervision, Methodology, Conceptualization. **Beáta Kerémi:** Writing – review & editing, Supervision, Formal analysis. **Orsolya Németh:** Writing – review & editing, Supervision, Methodology, Conceptualization.

## Ethical approval, complete ethics statement

No ethical approval was required for this systematic review with meta-analysis, as all data were already published in peer-reviewed journals. No patients were involved in the design, conducting, or interpretation of our study.

Review and/or approval by an ethics committee was not needed for this systematic review with meta-analysis, as all data were already published in peer-reviewed journals. No patients were involved in the design, conducting, or interpretation of our study.

Informed consent was not required for this study because for this systematic review with meta-analysis, as all data were already published in peer-reviewed journals. No patients were involved in the design, conducting, or interpretation of our study.

The datasets used in this study can be found in the full-text articles included in the systematic review and meta-analysis.

## Data availability statement

Data associated with your study has not been deposited into a publicly available repository, since all data is included in article/supp. material/referenced in article.

## Funding

No funding to declare.

## Declaration of competing interest

The authors declare that they have no known competing financial interests or personal relationships that could have appeared to influence the work reported in this paper.
