# Supplementary material for: Evaluating salivary MMP-8 as a biomarker for periodontal diseases: A systematic review and meta-analysis
Source: Heliyon. 2024 Nov 14;10(22):e40402. doi: 10.1016/j.heliyon.2024.e40402 (PMC11617734; doi:10.1016/j.heliyon.2024.e40402)
Supplement: Multimedia component 1 [file mmc1.docx]

**Supplementary data**

Supplementary Table 1 - PRISMA flowchart

| **Section and Topic** | **Item #** | **Checklist item** | **Location where item is reported** |
| --- | --- | --- | --- |
| **TITLE** | | |  |
| Title | 1 | Identify the report as a systematic review. | 1 |
| **ABSTRACT** | | |  |
| Abstract | 2 | See the PRISMA 2020 for Abstracts checklist. | 3 |
| **INTRODUCTION** | | |  |
| Rationale | 3 | Describe the rationale for the review in the context of existing knowledge. | 4 |
| Objectives | 4 | Provide an explicit statement of the objective(s) or question(s) the review addresses. | 5 |
| **METHODS** | | |  |
| Eligibility criteria | 5 | Specify the inclusion and exclusion criteria for the review and how studies were grouped for the syntheses. | 5 |
| Information sources | 6 | Specify all databases, registers, websites, organisations, reference lists and other sources searched or consulted to identify studies. Specify the date when each source was last searched or consulted. | 5 |
| Search strategy | 7 | Present the full search strategies for all databases, registers and websites, including any filters and limits used. | 5 |
| Selection process | 8 | Specify the methods used to decide whether a study met the inclusion criteria of the review, including how many reviewers screened each record and each report retrieved, whether they worked independently, and if applicable, details of automation tools used in the process. | 6 |
| Data collection process | 9 | Specify the methods used to collect data from reports, including how many reviewers collected data from each report, whether they worked independently, any processes for obtaining or confirming data from study investigators, and if applicable, details of automation tools used in the process. | 6 |
| Data items | 10a | List and define all outcomes for which data were sought. Specify whether all results that were compatible with each outcome domain in each study were sought (e.g. for all measures, time points, analyses), and if not, the methods used to decide which results to collect. | 6 |
|  | 10b | List and define all other variables for which data were sought (e.g. participant and intervention characteristics, funding sources). Describe any assumptions made about any missing or unclear information. | 6 |
| Study risk of bias assessment | 11 | Specify the methods used to assess risk of bias in the included studies, including details of the tool(s) used, how many reviewers assessed each study and whether they worked independently, and if applicable, details of automation tools used in the process. | 6 |
| Effect measures | 12 | Specify for each outcome the effect measure(s) (e.g. risk ratio, mean difference) used in the synthesis or presentation of results. | 6,7 |
| Synthesis methods | 13a | Describe the processes used to decide which studies were eligible for each synthesis (e.g. tabulating the study intervention characteristics and comparing against the planned groups for each synthesis (item #5)). | 6,7 |
|  | 13b | Describe any methods required to prepare the data for presentation or synthesis, such as handling of missing summary statistics, or data conversions. | 6,7 |
|  | 13c | Describe any methods used to tabulate or visually display results of individual studies and syntheses. | 6,7 |
|  | 13d | Describe any methods used to synthesize results and provide a rationale for the choice(s). If meta-analysis was performed, describe the model(s), method(s) to identify the presence and extent of statistical heterogeneity, and software package(s) used. | 6,7 |
|  | 13e | Describe any methods used to explore possible causes of heterogeneity among study results (e.g. subgroup analysis, meta-regression). | 6,7 |
|  | 13f | Describe any sensitivity analyses conducted to assess robustness of the synthesized results. | 6,7 |
| Reporting bias assessment | 14 | Describe any methods used to assess risk of bias due to missing results in a synthesis (arising from reporting biases). | 7 |
| Certainty assessment | 15 | Describe any methods used to assess certainty (or confidence) in the body of evidence for an outcome. | 7 |
| **RESULTS** | | |  |
| Study selection | 16a | Describe the results of the search and selection process, from the number of records identified in the search to the number of studies included in the review, ideally using a flow diagram. | 7 |
|  | 16b | Cite studies that might appear to meet the inclusion criteria, but which were excluded, and explain why they were excluded. | 7 |
| Study characteristics | 17 | Cite each included study and present its characteristics. | 7 |
| Risk of bias in studies | 18 | Present assessments of risk of bias for each included study. | 7 |
| Results of individual studies | 19 | For all outcomes, present, for each study: (a) summary statistics for each group (where appropriate) and (b) an effect estimate and its precision (e.g. confidence/credible interval), ideally using structured tables or plots. | 8 |
| Results of syntheses | 20a | For each synthesis, briefly summarise the characteristics and risk of bias among contributing studies. | 8,9 |
|  | 20b | Present results of all statistical syntheses conducted. If meta-analysis was done, present for each the summary estimate and its precision (e.g. confidence/credible interval) and measures of statistical heterogeneity. If comparing groups, describe the direction of the effect. | 8,9 |
|  | 20c | Present results of all investigations of possible causes of heterogeneity among study results. | 8,9 |
|  | 20d | Present results of all sensitivity analyses conducted to assess the robustness of the synthesized results. | 8,9 |
| Reporting biases | 21 | Present assessments of risk of bias due to missing results (arising from reporting biases) for each synthesis assessed. | 8 |
| Certainty of evidence | 22 | Present assessments of certainty (or confidence) in the body of evidence for each outcome assessed. | 9 |
| **DISCUSSION** | | |  |
| Discussion | 23a | Provide a general interpretation of the results in the context of other evidence. | 9-13 |
|  | 23b | Discuss any limitations of the evidence included in the review. | 12 |
|  | 23c | Discuss any limitations of the review processes used. | 12 |
|  | 23d | Discuss implications of the results for practice, policy, and future research. | 13 |
| **OTHER INFORMATION** | | |  |
| Registration and protocol | 24a | Provide registration information for the review, including register name and registration number, or state that the review was not registered. | 5 |
|  | 24b | Indicate where the review protocol can be accessed, or state that a protocol was not prepared. | 5 |
|  | 24c | Describe and explain any amendments to information provided at registration or in the protocol. | 5 |
| Support | 25 | Describe sources of financial or non-financial support for the review, and the role of the funders or sponsors in the review. | 14 |
| Competing interests | 26 | Declare any competing interests of review authors. | 14 |
| Availability of data, code and other materials | 27 | Report which of the following are publicly available and where they can be found: template data collection forms; data extracted from included studies; data used for all analyses; analytic code; any other materials used in the review. |  |

*From:*  Page MJ, McKenzie JE, Bossuyt PM, Boutron I, Hoffmann TC, Mulrow CD, et al. The PRISMA 2020 statement: an updated guideline for reporting systematic reviews. BMJ 2021;372:n71. doi: 10.1136/bmj.n71

For more information, visit: <http://www.prisma-statement.org/>

Supplementary Table 2 – Search key used in the systematic search

| Search key |
| --- |
| "((AMMP-8) OR (MMP-8) OR (MMP) OR (matrix metalloproteinase) OR (matrix metalloproteinase 8) OR "matrix metalloproteinase-8" OR (matrix metalloproteinases) OR (active matrix metalloproteinase) OR (active matrix metalloproteinase 8) OR "active matrix metalloproteinase-8" OR (salivary mmp-8) OR (mmp8)) AND ((periodontitis) OR (periodontal disease) OR (periodontal outcomes) OR (periodontal destruction) OR (gingivitis) OR (gingival inflammation))". |

Supplementary Table 3 - Basic characteristics of included studies (extended)

| **Study** | **Country** | **Sample size** | **mean age**  **Male:female ratio** | **Smoking habit** | **Definition of periodontitis and**  **gingivitis** |
| --- | --- | --- | --- | --- | --- |
| **Akbari et al. 2013** | India | P:100  G:100  H:50 | 30-39 years | 5 groups:  50 nonsmoker subjects with clinically healthy periodontium,  50 gingivitis‐nonsmokers  50 chronic periodontitis‐nonsmokers  50 gingivitis‐smokers  50 chronic periodontitis  group‐smokers | P: PI ≥2, GI ≥2, mean  OHI‐S ≥3 and CAL ≥5 mm.  G: PI ≥2, mean GI ≥2, mean OHI‐S ≥3 and no AL. |
| **Bostanci et al. 2021** | Turkey | P: 60  G: 31  H: 36 | female: male- 71:56  Age:  P: 39.6±5.7  G:33.1±5.9  H:33.7±6.7 | non-smoker individuals | P:  generalized aggressive periodontitis: CAL≥5 mm and PPD≥6 mm on≥8 teeth with radiographic bone loss≥30% of root length affecting at least≥3 teeth other than first molars and incisors.  generalized chronic periodontitis: CAL≥5 mm, PPD≥6 mm, and≥50% alveolar bone loss in at least two quadrants.  G: above 50% bleeding on probing scores, no CAL>2 mm or radiographic alveolar bone loss. |
| **Christodoulides et al. 2007** | United States of America | P:28  H:28 | ≥18  years of age | - | P:>30% of gingival sites with BOP, >20% probing depths ≥4 mm, and  >5% of interproximal sites with clinical attachment loss (CAL) of >2 mm, and evidence of radiographic bone loss |
| **Ebersole et al. 2013** | United States of America | P:50  H:30 | P: age: 43.0±10.8  female: 28%  H: age: 31.4±6.8  female: 46,7% | P: 28% tobacco use  H: 0% tobacco use | P: minimum of two affected teeth in each quadrant with each site having PD≥5 mm, CAL of≥3 mm, and BOP score of≥2 |
| **Gupta et al. 2015** | India | P:40  H:20 | age range of 35–55 years  Group I: 43.30±8.64, 10 male: 10 female  Group II: 42.80±8.02, 11 male: 9 female  Group III: 44.20±7.40, 14 male: 6 female | 3 groups:  group I: healthy  nonsmoking subjects, group II: nonsmoking patients with chronic periodontitis  group III: smoking patients with chronic periodontitis. | P: at least two or more interproximal  sites with clinical attachment level (CAL) of ≥4 mm, or two or  more interproximal sites with PPD of ≥5 mm, not on the same  tooth) |
| **Gursoy et al. 2010** | Finland | P:84  H:81 | periodontitis smoker: 48.6±5.3, 52.3% men  periodontitis non-smoker: 50.7±4.9, 67.5% men  control smoker: 44.4±4.3, 42.9% men  Control non-smoker: 48.6±5.7, 33.3% men | P:  smoker: 52.3%  non-smoker: 47.6%  H:  smoker: 17.2%  non-smoker: 81.5% | P: at least 14 teeth  with a probing pocket depth (PPD) ≥4 mm |
| **Lee et al. 2020** | South Korea | P:93  H:28 | 38 male (30.4%) and 87 female (69.6%)  age: H - 30.04±8.79  P- PS-I: 35.00±15.10  PS-II:49.21±16.92  PS-III: 58.17±14.40  PS-IV: 61.41±11.35 | smokers:  H: 0 (0.0%)  PS I: 0 (0.0%)  PS II: 2 (8.3%)  PS III:4 (17.4%)  PS IV: 1 (4.5%) | P: staging and grading based on [1] |
| **Miller et al. 2006** | United States of America | P:28  H:29 | H: age 43.1±7.2  41.4% male  P: age 45.4±8.5  42.9% male | H: 27.6% smoking  P: 33.3% smoking | P: probing, at least 20 percent of periodontal sites had probing depths (PD) of 4 mil-  limeters or greater, at least 5 percent of periodontal sites had interproximal clinical attachment loss (CAL) greater than 2 mm and radiographic bone loss was evident in posterior vertical bitewing films. |
| **Rai et al. 2008** | India | P:20  G:18  H:15 | Age  P: 35.3±9.6  G: 36.1±9.3  H: 35.1±8.7 | - | P: presence of moderate to advanced chronic periodontitis (at least 7 teeth with periodontal pockets deeper than 6 mm)  G: presence of generalized  gingival inflammation with bleeding on probing. |
| **Ramseier et al. 2009** | United States of America | P:49  G:32  H:18 | Mean age + males %  Group A  (healthy): 45 years, 56%  Group B  (gingivitis): 42 years, 41%  Group C  (mild chronic  periodontitis): 53 years, 39%  Group D  (moderate to  severe chronic  periodontitis): 50 years, 38 % | Smokers  Group A: 0%  Group B: 19%  Group C: 36%  Group D: 81% | P: at least four sites with evidence of radiographic bone loss, at least four sites with attachment loss >3 mm, and at  least four sites with PD >4 mm  G: <3 mm of attachment loss, no periodontal probing depth (PD) >4 mm, and no radiographic alveolar bone loss. BOP >20% |
| **Rangbulla et al. 2017** | India | P:30  H:20 | aged 18 to 45 years | P: non-smokers | P: at least four sites with evidence of radiographic bone loss, at least four sites with attachment loss >3 mm, and at  least four sites with PD >4 mm |
| **Umeizudike et al. 2022** | United Kingdom | P:67  G:63  H:59 | Females (n = 118)  represented 62.4% of the total study population (n = 189), while  the mean age of all participants was 40.4 ± 11.7 years (range  18–62 years). | non-smokers | P: interproximal PPD ≥5 mm at ≥8 teeth and BOP ≥30%.  G: mGI of ≥3.0 in ≥30% of sites, no sites with interproximal attachment loss or PPD ≥4 mm, and BOP ≥10% |
| **Zhang et al. 2021** | China | P:31  G:24  H:25 | Age+Gender (M/F)  P: 42.58±3.39, 17/14  G: 26.32±4.02, 11/13  H: 24.68±3.52, 12/13 | non-smokers | P: presence of interdental CAL≥5 mm, PD≥6 mm and radiographic bone loss extending to 2/3 of the root or beyond.  G: presence of bleeding on probing and BOP≥10%, PD≤3 mm, no clinical attachment loss, no radiographic bone loss. |
| **Keles et al. 2020** | Turkey | P:40  G:20  H:23 | aged 25-50 years (41 females and  42 males; mean age: 37.16 ± 5.96 years) | Non-smokers | G: PD ≤ 3 mm with BOP > 50% in the entire mouth as well as no clinical attachment loss or alveolar bone loss.  P: interdental CAL ≥ 5 mm, PD ≥ 6 mm and radiographic bone loss extending to the mid-third of the root or beyond. |
| **Mauramo et al. 2021** | Switzerland | P:116  H:86 | Mean age:  P: 48.2 (29–56)  H:42.9 (25–57)  Male/Female (%/%)  P:55/61 (47.4/52.6)  H:27/59 (31.4/68.6) | Never, n (%)  H: 52 (60.5)  P: 58 (50.0)  Former, n (%)  H: 21 (24.4)  P: 38 (32.8)  Current, n (%)  H: 13 (15.1)  P: 21 (17.2) | P: severe if ≥2 interproximal sites with CAL ≥6 mm and ≥ 1 interproximal sites with PPD ≥5 mm; moderate if ≥2 interproximal sites with CAL  ≥4 mm or ≥ 2 interproximal sites with PPD ≥5 mm; and mild if ≥2 interproximal sites with CAL ≥3 mm and ≥ 2 interproximal sites with  PPD ≥4 mm or ≥ 1 interproximal sites with PPD ≥5 mm. |
| **Nizam et al. 2014** | Turkey | P:18 (chronic periodontitis)  H:18 | P: GCP (10 males and 8 females; age 50.0* (45.0–54.0))  H:(11 males and 7 females; age 44.50* (39.50–52.50)) | Smoking (non-smoker/smoker/former smoker)  GCP: 8/8/2  H: 8/6/4 | P: had ≥4 teeth in each jaw with a probing depth (PD) of ≥5 mm, clinical attachment level (CAL) of  ≥4 mm, and ≥50 % alveolar bone loss at least in two quadrants. |
| **Noack et al. 2017** | Germany | P:20  G:20  H:19 | Male (%)  H: 3 (15.8)  G: 11 (55.0)  P: 12 (60.0)  Age (Mean)  H: 24.3 (23.6-26.4)  G: 24.6 (23.2-25.3)  P: 50.3 (39.1-57.6) | Non-smokers(%)  H: 19 (100.0)  G: 19 (95.0)  P: 13 (65.0)  Former smokers(%)  H: 0 (0.0)  G: 0 (0.0)  P: 3 (15.0)  Current smokers(%)  H: 0 (0.0)  G: 1 (5.0)  P: 4 (20.0) | P: interproximal clinical attachment loss of ≥ 5 mm in at least 30% of teeth present  G: mean GI ≥ 0.5 and no interproximal site with clinical attachment loss > 1 mm or PPD > 3 mm. |
| **Ozturk et al. 2021** | Turkey | P:37  G:21  H:22 | Age (mean-years)  PSIV: 41 ± 7.8  PSIII:44.7 ± 9.6  G: 30 ± 9.0  H: 31 ± 6.4  Females/Males  PSIII: 11/8  PSIV: 8/10  G: 11/10  H: 10/12 | Non-smokers | P:interdental CAL, detectable at minimum of 2 non-adjacent teeth. Periodontitis patients were further divided into Stage III and IV groups according to the new international classi-  fication of periodontitis which was based on severity and complexity of management P-Stage III group – subjects who had probing depth > 6 mm, vertical bone loss >  3 mm, Class II or III furcation involvement and moderate ridge defect. P-Stage IV group included subjects with masticatory dysfunction,  secondary occlusal trauma and posterior bite collapse  G: varying degrees of gingival inflammation, CAL < 2 mm, and no evidence of radiographic bone loss due to periodontitis. |
| **Ebersole et al. 2015** | United States of America | P:101  G:43  H:65 | Age (years; mean ± SD)  H: 28.2 ± 5.9  G:27.8 ± 4.5  P:42.0 ± 10.4  Female (%)  H: 60.0  G: 48.8 P: 32.7 | Current tobacco use (%)  H: 0  G: 0  P: 28.0 | P: BOP at >10% of sites, with >5% of sites with PPD ≥4 mm and CAL ≥ 2 mm.  G: BOP at ≥20% of sites, <3% of sites with PPD ≥ 4 mm, and no sites with CAL ≥ 2 mm. |

Abbreviations: P: periodontitis group, G: gingivitis group, M:male, F:female, H: healthy group, BOP: Bleeding on probing, PPD: Probing Pocket Depth,
CAL: Clinical Attachment Loss

Supplementary Table 4 - Risk of bias assessment for case-control studies

|  | **Selection** | | | | **Comparability** | **Exposure** | | | **All** |
| --- | --- | --- | --- | --- | --- | --- | --- | --- | --- |
| **Author, year** | **Is the case definition adequate?** | **Representativeness of the cases** | **Selection of Controls** | **Definition of Controls** | **Comparability of cases and controls on the basis of the design or analysis** | **Ascertainment of exposure** | **Same method of ascertainment for cases and controls** | **Non-Response rate** | **✵** |
| Christodoulies et al. 2007 | **✵** | **✵** | **✵** | **✵** | **✵✵** | **✵** | **✵** | **✵** | **9** |
| Akbari et al. 2015 | **✵** | **✵** |  | **✵** | **✵✵** | **✵** | **✵** | **✵** | **8** |
| Ebersole et al. 2013 | **✵** | **✵** |  | **✵** | **✵** | **✵** | **✵** | **✵** | **7** |
| Gupta et al. 2015 | **✵** | **✵** |  | **✵** | **✵** | **✵** | **✵** | **✵** | **7** |
| Gursoy et al. 2010 | **✵** | **✵** | **✵** | **✵** | **✵✵** | **✵** | **✵** | **✵** | **9** |
| Lee et al. 2020 | **✵** | **✵** |  | **✵** | **✵** | **✵** | **✵** | **✵** | **7** |
| Miller et al. 2006 | **✵** | **✵** | **✵** | **✵** | **✵✵** | **✵** | **✵** | **✵** | **9** |
| Nizam et al. 2014 | **✵** | **✵** | **✵** | **✵** | **✵** | **✵** | **✵** | **✵** | **8** |
| Rai et al. 2008 | **✵** | **✵** | **✵** | **✵** | **✵** | **✵** | **✵** | **✵** | **7** |
| Ramseier et al. 2009 | **✵** | **✵** | **✵** | **✵** | **✵** | **✵** | **✵** | **✵** | **8** |
| Rangbulla et al. 2017 | **✵** | **✵** |  | **✵** | **✵** | **✵** | **✵** | **✵** | **7** |
| Zhang et al. 2021 | **✵** | **✵** | **✵** | **✵** | **✵✵** | **✵** | **✵** | **✵** | **9** |
| Johnson et al. 2016 | **✵** | **✵** | **✵** | **✵** | **✵** | **✵** | **✵** | **✵** | **8** |
| Keles et al. 2020 | **✵** | **✵** |  | **✵** | **✵✵** | **✵** | **✵** | **✵** | **8** |
| Mauramo et al. 2021 | **✵** |  | **✵** | **✵** | **✵** | **✵** | **✵** | **✵** | **7** |
| Noack et al. 2017 | **✵** | **✵** |  | **✵** | **✵** | **✵** | **✵** | **✵** | **7** |
| Ozturk et al. 2021 | **✵** | **✵** | **✵** | **✵** | **✵✵** | **✵** | **✵** | **✵** | **9** |
| Ebersole et al. 2015 | **✵** | **✵** | **✵** | **✵** | **✵** | **✵** | **✵** | **✵** | **8** |

Supplementary Table 5 - Risk of bias assessment for cohort studies

|  | **Selection** | | | | **Comparability** | **Outcome** | | | **All** |
| --- | --- | --- | --- | --- | --- | --- | --- | --- | --- |
| **Authoryear** | **Representativeness of the exposed cohort** | **Selection of the non- exposed cohort** | **Ascertainment of exposure** | **Demonstration that outcome of interest was not present at start of study** | **Comparability of cohorts on the basis of the design or analysis** | **Assessment of outcome** | **Was follow-up long enough for outcomes to occur?** | **Adequacy of follow up of cohorts** |  |
| Bostanci et al. 2021 | **✵** | **✵** | **✵** | **✵** | **✵✵** | **✵** | **✵** | **✵** | **9** |
| Umeizudike et al. 2022 | **✵** | **✵** | **✵** | **✵** | **✵✵** | **✵** | **✵** | **✵** | **9** |


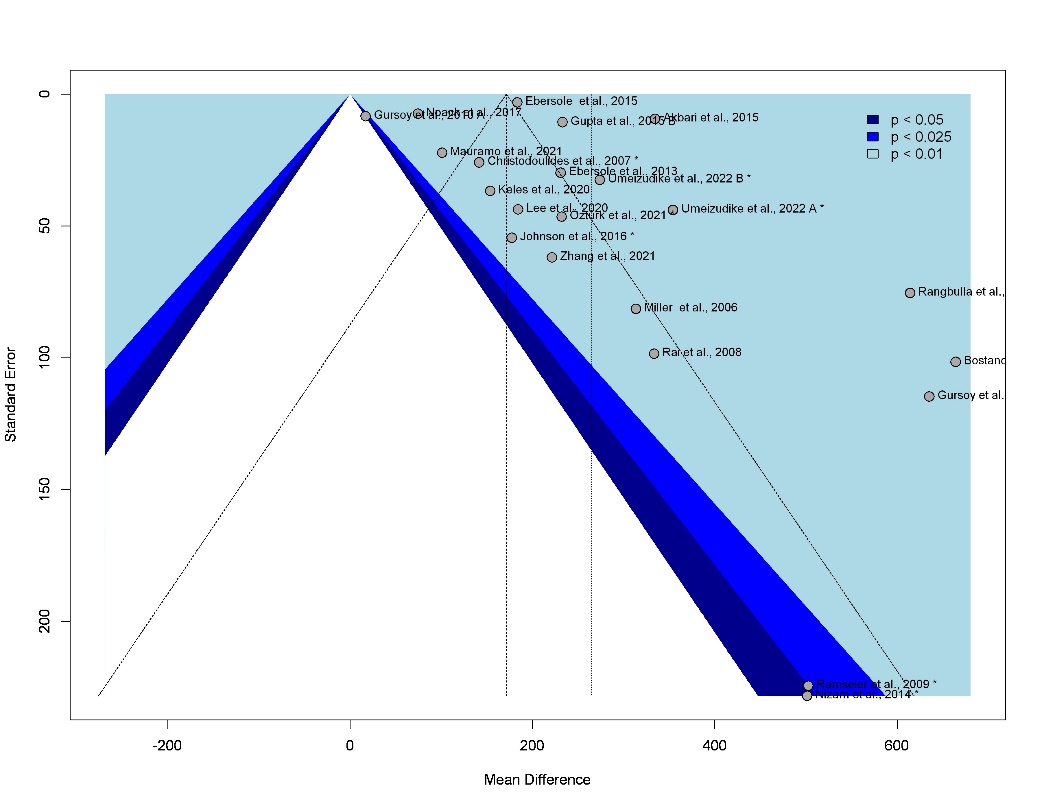


Supplementary Figure 1 - Periodontitis population funnel plot – all methods, Linear regression test of funnel plot asymmetry:
Test result: t = 0.90, df = 20, p-value = 0.3784


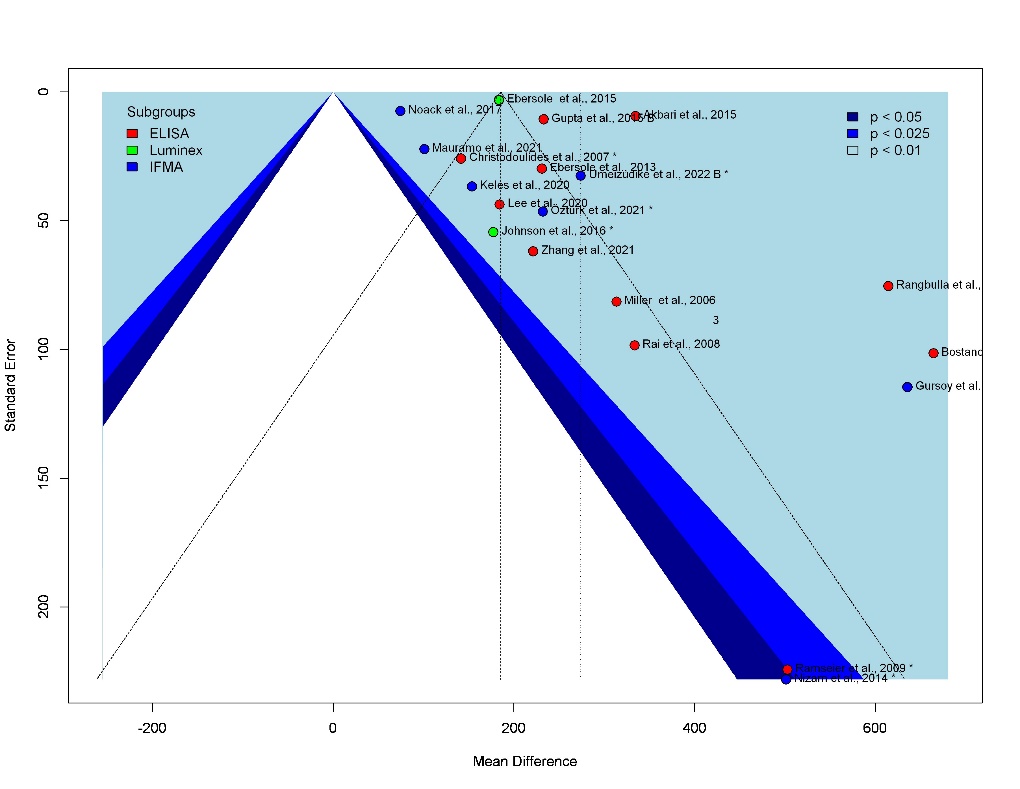


Supplementary Figure 2 - Periodontitis population funnel plot – subgroups ar marked with different colours, Gursoy et al. and Umeizuidike represented by IFMA population


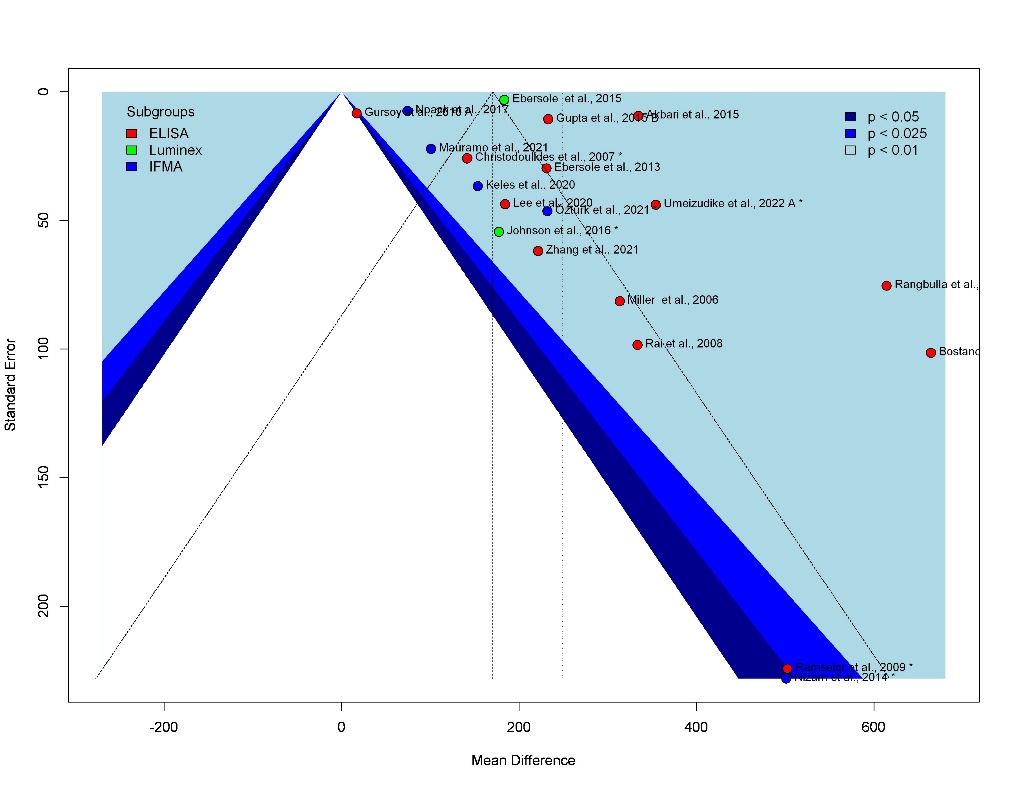


Supplementary Figure 3 - Periodontitis population funnel plot – subgroups are marked with different colours, Gursoy et al. and Umeizuidike represented by ELISA population


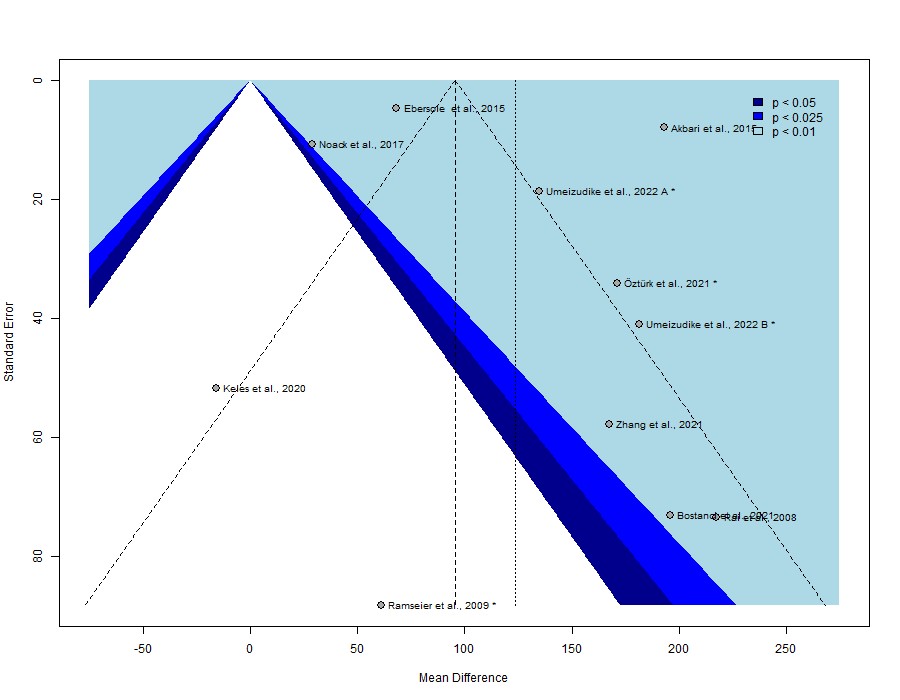


Supplementary Figure 4 - Gingivitis funnel plot – all methods,
Linear regression test of funnel plot asymmetry
Test result: t = 0.68, df = 9, p-value = 0.5111


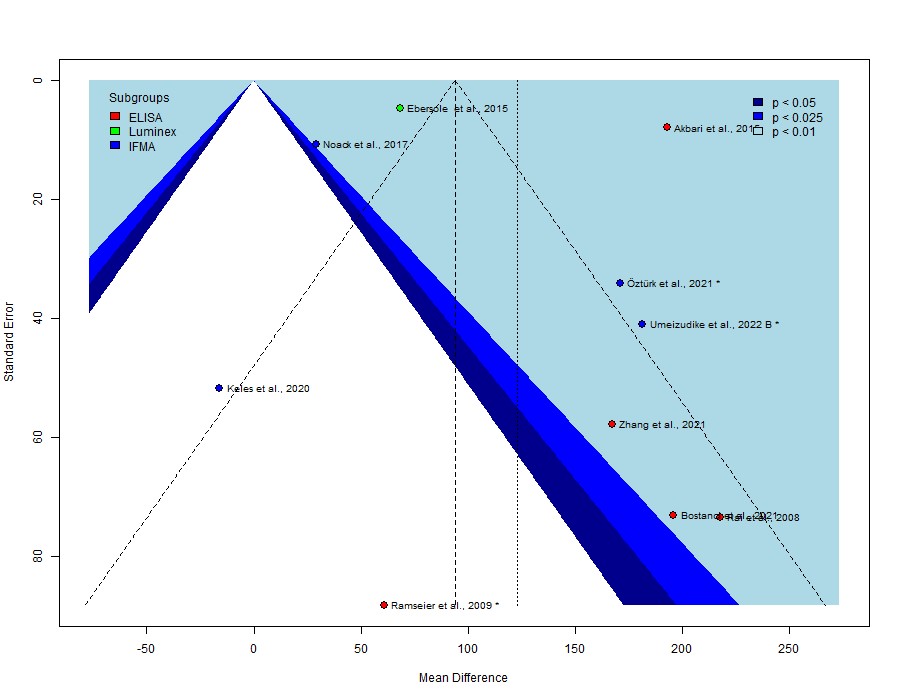


Supplementary Figure 5 - Gingivitis population funnel plot – subgroups are makred with different colours, Umeizuidike is represented by IFMA population


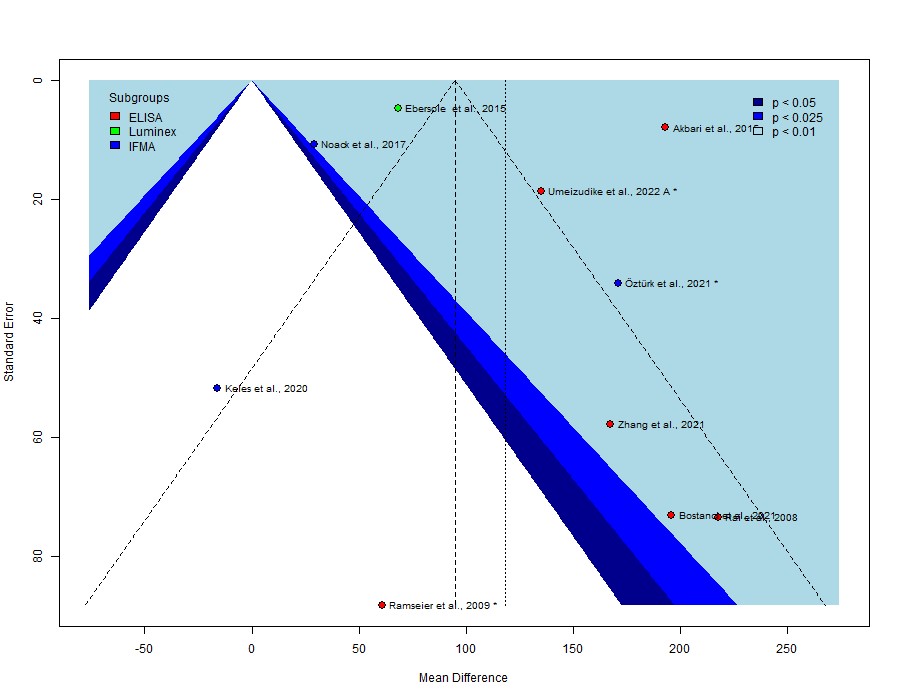


Supplementary Figure 6 - Gingivitis population funnel plot – subgroups are makred with different colours, Umeizuidike is represented by ELISA population


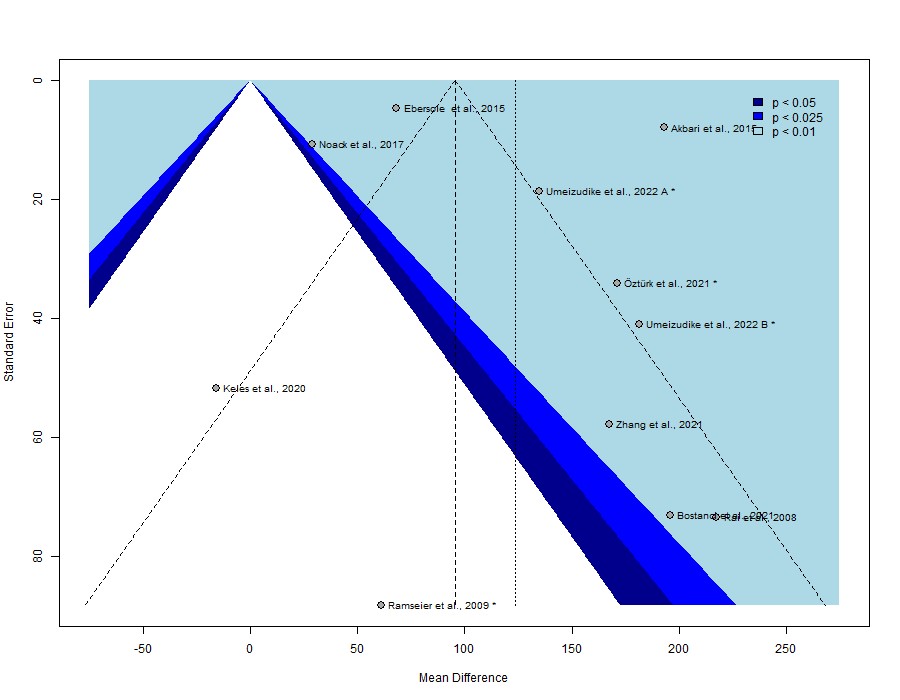


Supplemnetary Figure 7 - Periodontitis compared to gingivitis - funnel plot - all methods


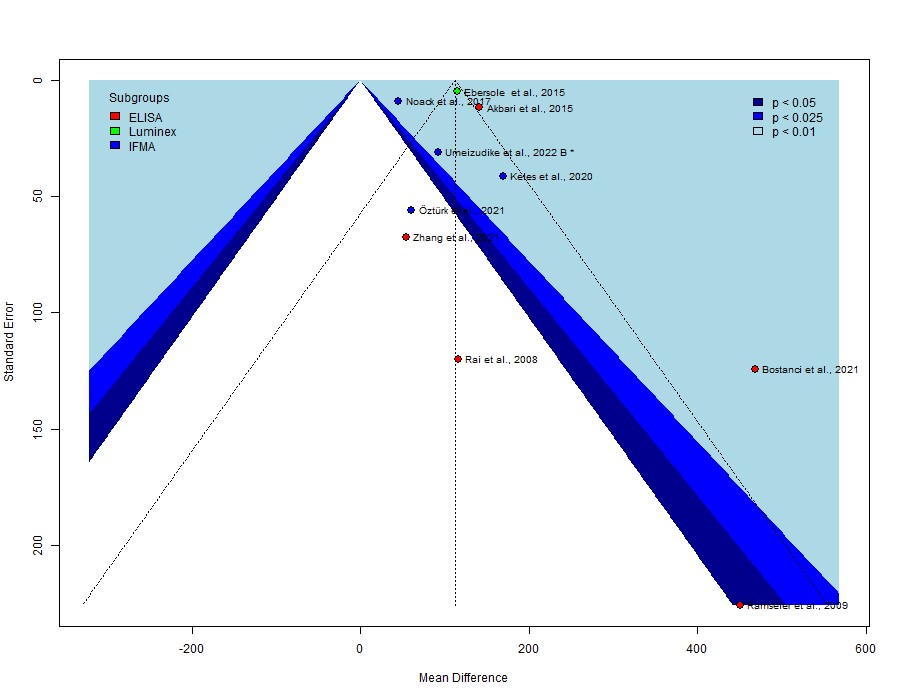


Supplemnetary Figure 8 - Periodontitis compared to gingivitis - funnel plot, subgroups are makred with different colours, Umeizuidike et al represented with IFMA popultaion


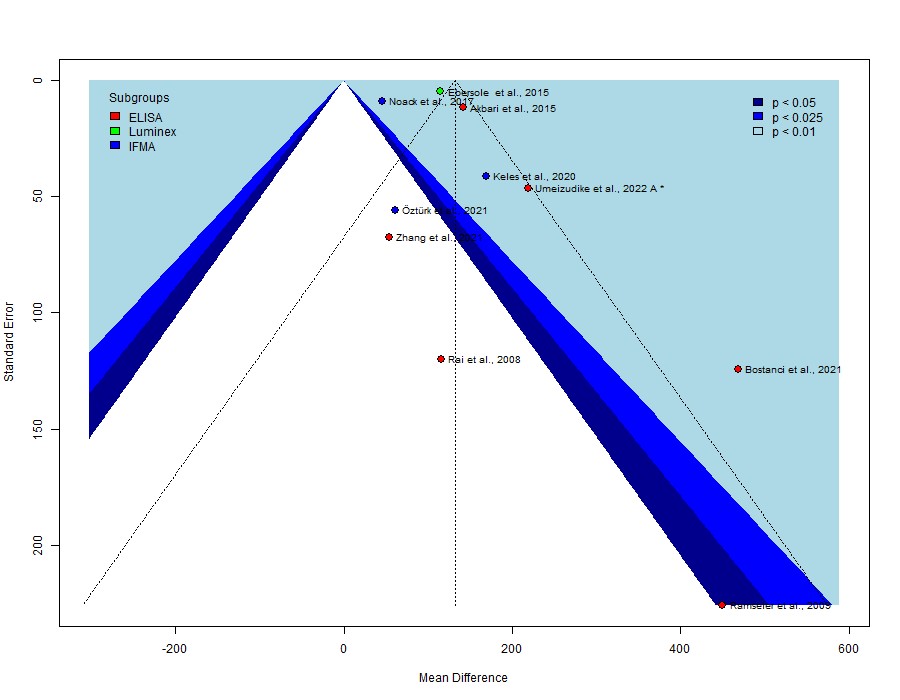


Supplemnetary Figure 9 - Periodontitis compared to gingivitis - funnel plot, subgroups are makred with different colours Umeizuidike et al represented with IFMA popultaion

Supplementary Table 6 - Table for statistical data, periodontitis group

| **Study_est** | **N_e** | **Mean_e** | **Mean_e_est** | **SD_e** | **SD_e_est** | **Median_e** | **Q1_e** | **Q3_e** | **Min_e** | **Max_e** | **N_c** | **Mean_c** | **Mean_c_est** | **SD_c** | **SD_c_est** | **Median_c** | **Q1_c** | **Q3_c** | **Min_c** | **Max_c** | |
| --- | --- | --- | --- | --- | --- | --- | --- | --- | --- | --- | --- | --- | --- | --- | --- | --- | --- | --- | --- | --- | --- |
| **Christodoulides et al., 2007 *** | 28 |  | 222.48 |  | 105.14 | 185.00 | 170.00 | 305.00 |  |  | 28 |  | 80.89 |  | 87.62 | 80.00 | 25.00 | 137.50 |  | |  |
| **Lee et al., 2020** | 93 | 419.19 |  | 267.580 |  |  |  |  |  |  | 28 | 235.01 |  | 178.71 |  |  |  |  |  | |  |
| **Zhang et al., 2021** | 31 | 657.10 |  | 279.800 |  |  |  |  |  |  | 25 | 435.80 |  | 180.60 |  |  |  |  |  | |  |
| **Ebersole et al., 2012** | 50 | 283.47 |  | 203.470 |  |  |  |  |  |  | 30 | 52.63 |  | 40.62 |  |  |  |  |  | |  |
| **Gupta et al., 2015 B** | 40 | 407.00 |  | 59.283 |  |  |  |  |  |  | 20 | 174.17 |  | 22.40 |  |  |  |  |  | |  |
| **Miller et al., 2006** | 28 | 408.60 |  | 423.300 |  |  |  |  |  |  | 29 | 95.10 |  | 80.10 |  |  |  |  |  | |  |
| **Rai et al., 2008** | 20 | 428.60 |  | 432.400 |  |  |  |  |  |  | 15 | 95.20 |  | 70.20 |  |  |  |  |  | |  |
| **Akbari et al., 2015** | 100 | 407.94 |  | 91.101 |  |  |  |  |  |  | 50 | 73.51 |  | 15.26 |  |  |  |  |  | |  |
| **Ramseier et al., 2009 *** | 49 | 626.39 |  | 1513.248 |  |  |  |  |  |  | 18 |  | 123.84 |  | 253.97 | 23.60 | 2.50 | 322.50 |  | |  |
| **Rangbulla et al., 2017** | 30 | 672.18 |  | 411.000 |  |  |  |  |  |  | 20 | 57.95 |  | 31.64 |  |  |  |  |  | |  |
| **Bostanci et al., 2021** | 60 | 740.70 |  | 783.000 |  | 439.80 |  |  |  |  | 36 | 76.50 |  | 53.70 |  | 65.80 |  |  |  | |  |
| **Noack et al., 2017** | 20 | 100.35 |  | 15.820 |  | 105.42 | 98.28 | 108.43 |  |  | 19 | 26.11 |  | 28.40 |  | 10.47 | 3.09 | 39.74 |  | |  |
| **Mauramo et al., 2021** | 116 | 225.84 |  | 183.447 |  |  |  |  |  |  | 86 | 125.00 |  | 132.10 |  |  |  |  |  | |  |
| **Keles et al., 2020** | 40 | 779.32 |  | 87.260 |  |  |  |  |  |  | 23 | 625.74 |  | 163.10 |  |  |  |  |  | |  |
| **Öztürk et al., 2021 *** | 37 | 273.20 |  | 276.512 |  |  |  |  |  |  | 22 |  | 41.30 |  | 45.03 | 41.86 | 12.40 | 69.77 |  | |  |
| **Umeizudike et al., 2022 B *** | 67 | 569.54 |  | 95.410 |  | 589.29 | 482.14 | 653.57 |  |  | 59 |  | 295.62 |  | 233.03 | 267.86 | 153.57 | 460.71 |  | |  |
| **Nizam et al., 2014 *** | 18 |  | 1169.87 |  | 601.59 | 1149.30 | 798.80 | 1556.80 |  |  | 18 |  | 668.59 |  | 758.41 | 674.80 | 188.40 | 1144.00 |  | |  |
| **Gursoy et al., 2010 B** | 84 | 967.45 |  | 1023.853 |  |  |  |  |  |  | 81 | 332.09 |  | 231.57 |  |  |  |  |  | |  |
| **Johnson et al., 2016 *** | 31 |  | 244.47 |  | 292.73 | 129.80 |  |  | 19.1 | 1223.1 | 10 |  | 67.24 |  | 44.93 | 51.90 |  |  | 19.67 | | 157.96 |
| **Ebersole et al., 2015** | 101 | 314.10 |  | 25.500 |  |  |  |  |  |  | 65 | 130.70 |  | 14.50 |  |  |  |  |  |  |  |

Supplementary Table 7 - Results used for statistical analysis, gingivitis patients

| **Study_est** | **N_e** | **Mean_e** | **Mean_e_est** | **SD_e** | **SD_e_est** | **Median_e** | **Q1_e** | **Q3_e** | **N_c** | **Mean_c** | **Mean_c_est** | **SD_c** | **SD_c_est** | **Median_c** | **Q1_c** | **Q3_c** |
| --- | --- | --- | --- | --- | --- | --- | --- | --- | --- | --- | --- | --- | --- | --- | --- | --- |
| **Ramseier et al., 2009 *** | 32 |  | 184.68 |  | 366.02 | 54.10 | 1.00 | 473.90 | 18 |  | 123.84 |  | 253.97 | 23.60 | 2.50 | 322.50 |
| **Zhang et al., 2021** | 24 | 603.20 |  | 220.700 |  |  |  |  | 25 | 435.80 |  | 180.60 |  |  |  |  |
| **Akbari et al., 2015** | 100 | 266.56 |  | 77.272 |  |  |  |  | 50 | 73.51 |  | 15.26 |  |  |  |  |
| **Bostanci et al., 2021** | 31 | 272.40 |  | 403.200 |  | 150.50 |  |  | 36 | 76.50 |  | 53.70 |  | 65.80 |  |  |
| **Rai et al., 2008** | 18 | 312.80 |  | 301.800 |  |  |  |  | 15 | 95.20 |  | 70.20 |  |  |  |  |
| **Keles et al., 2020** | 20 | 609.77 |  | 174.130 |  |  |  |  | 23 | 625.74 |  | 163.10 |  |  |  |  |
| **Noack et al., 2017** | 20 | 55.04 |  | 38.170 |  | 57.54 | 12.97 | 88.66 | 19 | 26.11 |  | 28.40 |  | 10.47 | 3.09 | 39.74 |
| **Öztürk et al., 2021 *** | 21 |  | 212.40 |  | 150.12 | 212.40 | 117.83 | 306.98 | 22 |  | 41.30 |  | 45.03 | 41.86 | 12.40 | 69.77 |
| **Umeizudike et al., 2022 B *** | 63 |  | 477.09 |  | 219.15 | 503.57 | 321.43 | 610.71 | 59 |  | 295.62 |  | 233.03 | 267.86 | 153.57 | 460.71 |
| **Ebersole et al., 2015** | 43 | 199.00 |  | 29.100 |  |  |  |  | 65 | 130.70 |  | 14.50 |  |  |  |  |


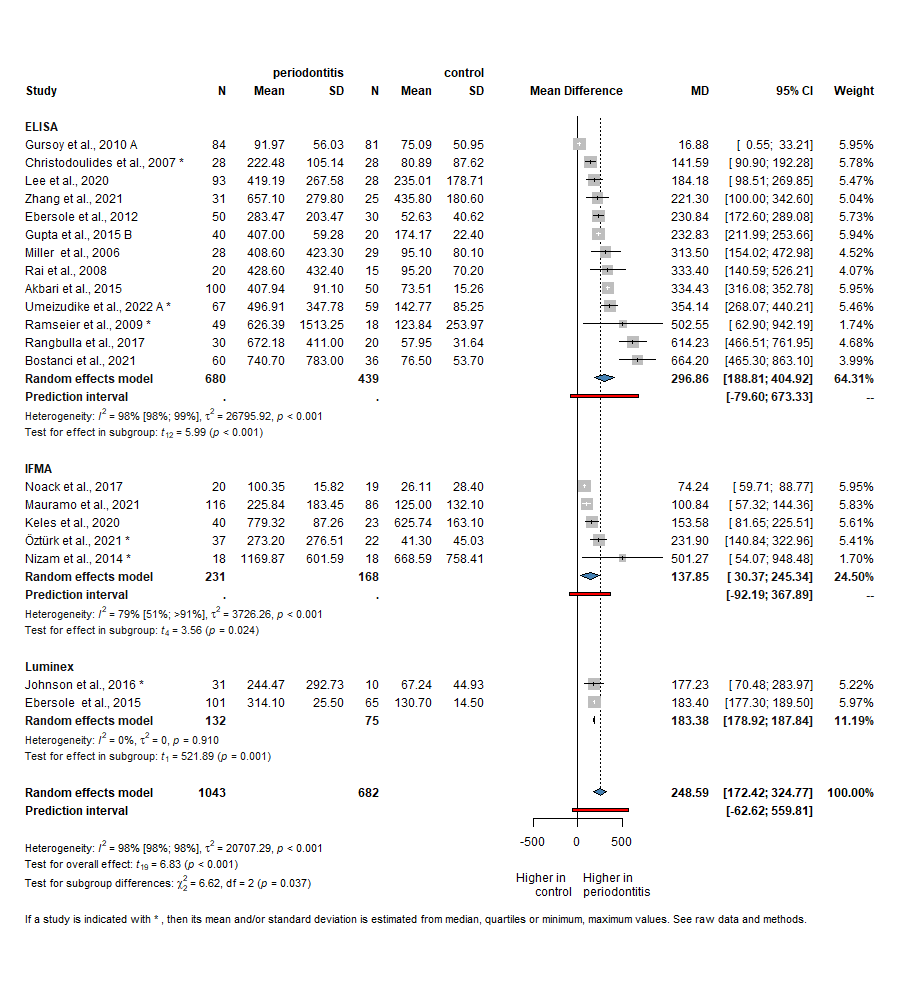
Supplementary Figure 10 - Mean difference of MMP-8 level results of periodontitis patients compared to healthy population (ELISA results used if both ELISA and IFMA results were available)


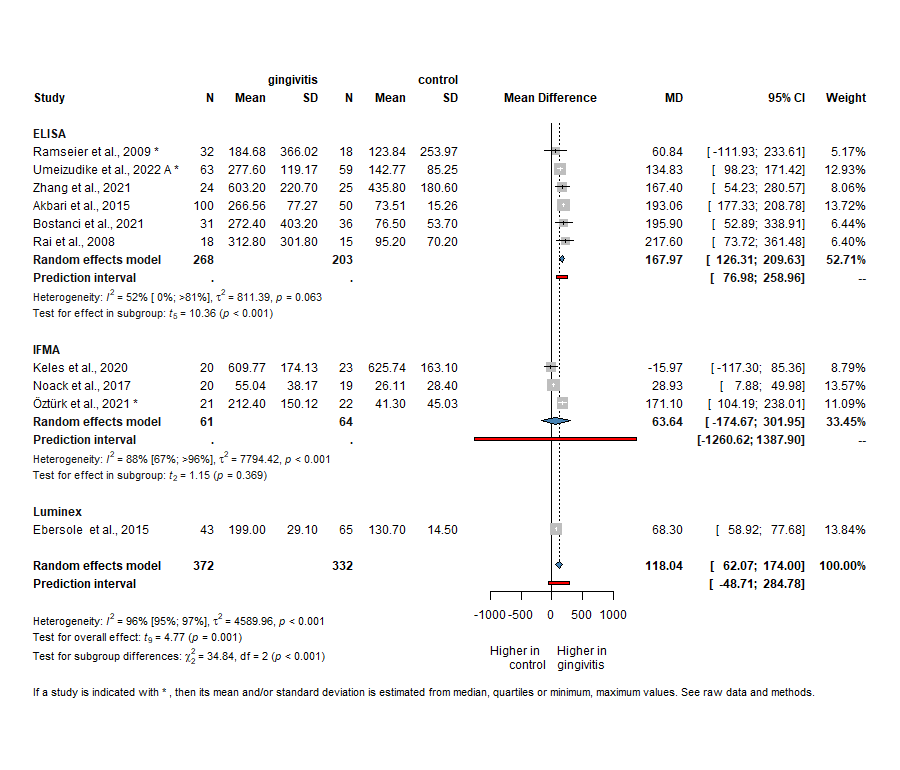


Supplementary Figure 11 - Mean difference of MMP-8 level results of gingivitis patients compared to healthy population (ELISA results used if both ELISA and IFMA results were available)


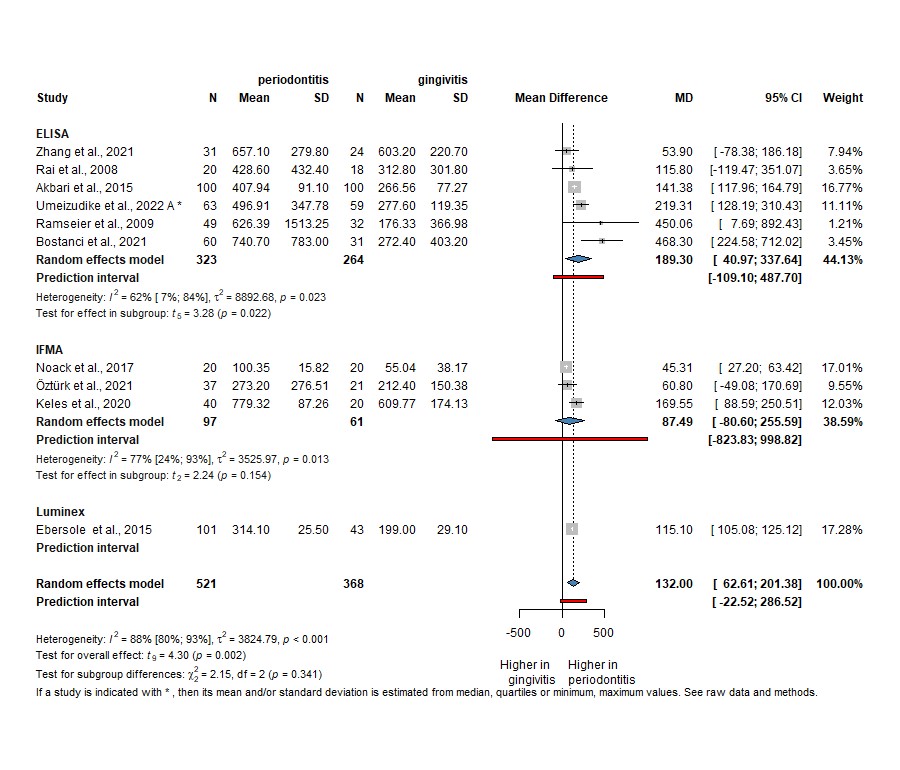


Supplementary Figure 12 - Mean difference of MMP-8 level results of periodontitis patients compared to gingivitis population (ELISA results used if both ELISA and IFMA results were available)

Supplementary Table 8 – GRADE Assessment of outcomes

| **Certainty assessment** | | | | | | | **№ of patients** | | **Effect** | **Certainty** |  |
| --- | --- | --- | --- | --- | --- | --- | --- | --- | --- | --- | --- |
| **№ of studies** | **Study design** | **Risk of bias** | **Inconsistency** | **Indirectness** | **Imprecision** | **Other considerations** | **[intervention]** | **[comparison]** | **Absolute (95% CI)** |  |  |
| **Periodontitis MMP-8** | | | | | | | | | | | |
| 20 | observational studies | not serious | not serious | not serious | not serious | strong association | 1043 | 682 | MD **273.26 ng/ml higher** (CI: 194.42 to 352.1) | ⨁⨁⨁◯ Moderate |  |
| **Gingivitis MMP-8** | | | | | | | | | | | |
| 10 | observational studies | not serious | not serious | not serious | not serious | strong association | 372 | 332 | MD **122.82 ng/ml higher** (CI: 64.19 to 181.45 ) | ⨁⨁⨁◯ Moderate |  |
| **Gingivitis vs perio** | | | | | | | | | | | |
| 10 | observational studies | not serious | not serious | not serious | not serious | strong association | 521 | 368 | MD **112,04 ng/ml higher** (CI: 56,16 to 167,92) | ⨁⨁⨁◯ Moderate |  |

**CI:** confidence interval; **MD:** mean difference
